# SUMO targeting of a stress-tolerant Ulp1 SUMO protease

**DOI:** 10.1371/journal.pone.0191391

**Published:** 2018-01-19

**Authors:** Jennifer Peek, Catherine Harvey, Dreux Gray, Danny Rosenberg, Likhitha Kolla, Reuben Levy-Myers, Rui Yin, Jonathan L. McMurry, Oliver Kerscher

**Affiliations:** 1 Biology Department, The College of William & Mary, Williamsburg, Virginia, United States of America; 2 Department of Molecular & Cellular Biology, Kennesaw State University, Kennesaw, Georgia, United States of America; Chinese University of Hong Kong, CHINA

## Abstract

SUMO proteases of the SENP/Ulp family are master regulators of both sumoylation and desumoylation and regulate SUMO homeostasis in eukaryotic cells. SUMO conjugates rapidly increase in response to cellular stress, including nutrient starvation, hypoxia, osmotic stress, DNA damage, heat shock, and other proteotoxic stressors. Nevertheless, little is known about the regulation and targeting of SUMO proteases during stress. To this end we have undertaken a detailed comparison of the SUMO-binding activity of the budding yeast protein Ulp1 (ScUlp1) and its ortholog in the thermotolerant yeast *Kluyveromyces marxianus*, KmUlp1. We find that the catalytic UD domains of both ScUlp1 and KmUlp1 show a high degree of sequence conservation, complement a *ulp1Δ* mutant *in vivo*, and process a SUMO precursor *in vitro*. Next, to compare the SUMO-trapping features of both SUMO proteases we produced catalytically inactive recombinant fragments of the UD domains of ScUlp1 and KmUlp1, termed ScUTAG and KmUTAG respectively. Both ScUTAG and KmUTAG were able to efficiently bind a variety of purified SUMO isoforms and bound immobilized SUMO1 with nanomolar affinity. However, KmUTAG showed a greatly enhanced ability to bind SUMO and SUMO-modified proteins in the presence of oxidative, temperature and other stressors that induce protein misfolding. We also investigated whether a SUMO-interacting motif (SIM) in the UD domain of KmULP1 that is not conserved in ScUlp1 may contribute to the SUMO-binding properties of KmUTAG. In summary, our data reveal important details about how SUMO proteases target and bind their sumoylated substrates, especially under stress conditions. We also show that the robust pan-SUMO binding features of KmUTAG can be exploited to detect and study SUMO-modified proteins in cell culture systems.

## Introduction

Sumoylation is the posttranslational modification of cellular proteins with the **s**mall **u**biquitin-like **mo**difier protein SUMO and is analogous to the modification of proteins with ubiquitin (ubiquitination). Sumoylation relies on the step-wise interactions of SUMO E1 (activating), SUMO E2 (conjugating) and SUMO E3 (ligating) enzymes, culminating in the addition of conjugation-competent SUMO to lysine residues of substrate proteins [1,2]. These lysine residues may be part of a sumoylation consensus motif (ΨKxD/E) of the target protein [3]. Yeast cells express one SUMO protein (Smt3) and mammalian cells express 3 (SUMO1, SUMO2 and SUMO3). A 4^th^ mammalian paralog of SUMO, SUMO4, is not processed and conjugated to proteins under physiological conditions. Smt3 in yeast and SUMO2/3 in mammalian cells can also form polymers or chains. SUMO chains are formed via sequential interconnection of SUMO monomers using internal lysine residues. Unlike proteins modified with ubiquitin chains, SUMO chain–modified proteins are not directly targeted to the proteasome. However, polysumoylation can lead to ubiquitination via SUMO-targeted ubiquitin ligases (STUbLs) and proteins carrying hybrid SUMO/ubiquitin chains may be targeted for proteasomal degradation [4–8]. Thousands of SUMO-modified proteins have been identified but the functional relevance of SUMO modification is often difficult to elucidate [9]. Based on research over the last 20 years, sumoylation of many key regulatory proteins plays a pivotal role in cell cycle regulation, nuclear transport, the DNA damage response, and chromosome segregation and affects various cellular processes [10].

The accumulation of sumoylated proteins in the cell is in part counterbalanced by dedicated SUMO-specific cysteine proteases that cleave SUMO off proteins that have been sumoylated. Much of what we know about SUMO protease function, activity, and specificity was initially identified in budding yeast S*accharmyces cerevisiae* (Sc) [11–14]. Budding yeast cells express two SUMO proteases of the Ulp family, ScUlp1 and ScUlp2. ScUlp1 is required for processing of the SUMO precursor and several nuclear and cytosolic SUMO-modified proteins [11]. In contrast, ScUlp2 preferentially cleaves SUMO chains and desumoylates a handful of nuclear substrates [15–17]. Differences in substrate specificity are due to specific domains of ScUlp1 and ScUlp2 that affect their localization within the cell and their ability to act on SUMO chains, monomeric SUMO, and sumoylated proteins. For example, karyopherin-interacting domains enrich ScUlp1 at the nuclear envelope, restrict its access to the nuclear interior, and control its cell cycle-regulated nuclear egress to interact with sumoylated septins at the bud neck of dividing cells [14,18,19]. ScUlp2, on the other hand, is targeted to the nuclear interior and carries SUMO-interacting motifs (SIMs) that may facilitate its interaction with SUMO chains [15]. SIMs usually consist of 3–4 hydrophobic residues (usually Val or Ile), that are often juxtaposed to a negatively charged amino acid (e.g. Glu or Asp). SIMs been found in many eukaryotic proteins and have in several cases been confirmed to promote the interaction with SUMO, SUMO chains, and sumoylated proteins [20].

Distinct cellular functions have been attributed to Ulp1 and Ulp2 activity. First, Ulp1 is an essential SUMO protease who’s role in genome maintenance and cell cycle progression is still not fully understood [11,21]. Impairment of Ulp1’s SUMO processing activity also adversely affects many other cellular processes such as ribosome biogenesis, DNA damage response, cellular DNA repair processes, the processing and export of the 60S pre-ribosomal particle, nucleus–cytoplasm trafficking, and cell viability (reviewed in [22]). Second, impairment of Ulp2 SUMO processing activity results in the accumulation of high-molecular weight polySUMO chains. PolySUMO chains are normally formed when cells are exposed to proteotoxic and genotoxic stressors. It has been hypothesized that this rapid increase of poly-sumoylation, termed the SUMO-stress response (SSR), may be linked to a wave of transcription-coupled sumoylation of mostly chromatin-associated proteins [23–25]). Therefore, Ulp2 may be required to counteract the persistence of polySUMO chains that may interfere with restarting normal transcriptional programs [17,23,26]. Ulp1 has also been linked to the cellular stress response. For example, Ulp1 is sequestered in the nucleolus in response to alcohol stress but not other stressors [27]. Additionally, upon mild oxidative stress exposure Ulp1 forms protective dimers, to prevent the irreversible oxidation of its catalytic cysteine residues [28]. This inactivation of SUMO proteases during acute cellular stress likely contributes to the formation of SUMO chains, suggesting an important role for the SSR. How the SSR and the accumulation of SUMO chains is reversed is not entirely clear but it may involve the SUMO-chain-mediated activation of STUbLs, the resulting degradation of SUMO ligases such as Siz, and SUMO-chain specific SUMO proteases [29,30].

Yeast Ulp1 and Ulp2 are evolutionarily conserved in the form of at least 6 distinct SENP proteases in mammalian cells. These SUMO proteases are differentially distributed to the nucleoplasm, nucleolus, nuclear envelope, cytosol, nuclear bodies, and to mitochondria (reviewed in [22,31,32]). SENP1 and SENP2 are most similar to Ulp1 and are, together with SENP5, able to process the precursors of SUMO1, 2 and 3. SENP6 and SENP7 are most similar to Ulp2 and are also involved in SUMO chain editing. Functionally, SENP proteases play a role in ribosome biogenesis and regulate several other critically important nuclear activities including transcription, genome maintenance, recombination, and chromosome segregation. Clinically relevant dysregulation or overexpression of the SUMO protease SENP1 plays a role in cancer development [33,34]. Additionally, cell culture and animal models indicate that SENP1 and SENP2 prevent apoptosis of neuronal cells [35,36].

How do SUMO proteases target their sumoylated substrates? Access of SUMO proteases to sumoylated proteins seems to be restricted by their subcellular localization. SUMO proteases also possess sequence features that facilitate their interaction with SUMO. Structural studies show that the catalytic domain of these SUMO proteases includes a SUMO-binding surface (SBS) that makes multiple contacts with SUMO [37]. In yeast Ulp1 and the related mammalian SENP1 and SENP2, the SBS is configured to allow binding and processing of all SUMO isoforms and their conjugates. In contrast, the SBS in SENP6 and SENP7 is altered to preferentially accommodate chain-forming SUMO2/3 [38]. Ulp2, which is most similar to SENP6/7, preferentially binds and processes chains of more than three SUMOs from the distal end, leaving a tail of two SUMOs attached to its substrates [16]. SIMs that could promote the interaction with sumoylated proteins and SUMO chains have been predicted in Ulp2, SENP1, SENP2, SENP6 and SENP7 but not in Ulp1, SENP3 and SENP5 [22]. In all cases, these SIMs are part of the non-catalytic regions but not the UD domain of these SUMO proteases. There is currently no published evidence linking SIMs in SUMO proteases to their activity or specificity.

We previously investigated how the budding yeast SUMO protease ScUlp1 that resides at the inner face of the nuclear pore complex targets sumoylated septins at the bud-neck of dividing cells [19]. We found that the substrate-targeting features of Ulp1 are restricted to its carboxy-terminal catalytic UD domain. Interestingly, we also determined that the UD domain of Ulp1 with the catalytic cysteine (C580) replaced by a serine, traps sumoylated proteins. For simplicity we will refer to this carboxy-terminal SUMO-trapping Ulp1(C580S) fragment as UTAG (short for UD TAG). The budding yeast–derived UTAG (ScUTAG) is 218 amino acids in length and can readily be expressed as a recombinant fusion protein in bacterial cells. When expressed in yeast cells, ScUTAG localizes to the septin ring and the nucleus of G2/M-arrested cells. The recombinant protein is able to pull down sumoylated proteins from yeast protein extract and interact with SUMO chains. We previously found that the ScUTAG avidly binds to immobilized SUMO1 with a Kd of ~12.8nM, which is about 200 times stronger than the interaction of SUMO with a SIM.

This remarkable affinity of the ScUTAG for SUMO raised the possibility that the UTAG could be a useful reagent to identify important mitotic targets of Ulp1 and aid our studies of how SUMO proteases interact with SUMO and sumoylated proteins. In our search for features that could enhance SUMO binding and processing by SUMO proteases, we investigated a variant of ScUlp1 from the thermotolerant yeast strain *Kluyveromyces marxianus*. KmUlp1 contains a *bona fide* surface-exposed SIM sequence in the UD domain that is not conserved in ScUlp1. In the current study we cloned the KmUD to generate KmUTAG. A previous study showed that recombinant proteins derived from *Kluyveromyces marxianus* exhibit superior stability when exposed to high temperature and chemical insults relative to their budding yeast orthologs [39]. When compared to ScUTAG, we found that KmUTAG has greatly enhanced pan-SUMO-binding properties under conditions that include oxidative stress, heat stress, and protein denaturing reagents. Unexpectedly, replacement of the putative SIM in KmUTAG, greatly reduced its ability to interact with immobilized SUMO even at ambient temperature. However, whether this putative SIM plays a physiological role in SUMO binding is not yet clear. Overall, we find that the binding of KmUTAG to SUMO is faster than ScUTAG and that KmUTAG is retained longer on immobilized SUMO. Finally, we show that KmUTAG is a useful reagent to identify sumoylated proteins and that KmUTAG fused to a fluorescent protein can be used to detect sumoylated proteins in mammalian tissue culture cells. In summary, the KmUTAG is a unique reagent for the study of SUMO processing and sumoylation, and it may allow us to address fundamental questions about the roles of SUMO under stress.

## Results

### A Ulp1 SUMO protease from *K*. *marxianus*, a thermotolerant yeast strain

SUMO proteases are pivotal components of the sumoylation cycle in all eukaryotes but the question of how these enzymes target specific substrates, especially those involved in cell cycle progression, remains largely unanswered. Our quest to identify determinants of SUMO-targeting in SUMO proteases led us to analyze a Ulp1 protease from *Kluyveromyces marxianus* (*K*. *marxianus* or *Km*). *K*. *marxianus* is a thermotolerant yeast strain and proteins isolated from this yeast show exceptional tolerance to heat and chemical stressors [39]. We reasoned that this feature of *K*. *marxianus* would extend to proteins of the sumoylation cycle, including Ulp1, and that KmUlp1 would possess sequence features allowing it to target, bind, and process SUMO and SUMO-modified proteins under otherwise unfavorable conditions.

**Fig 1:**
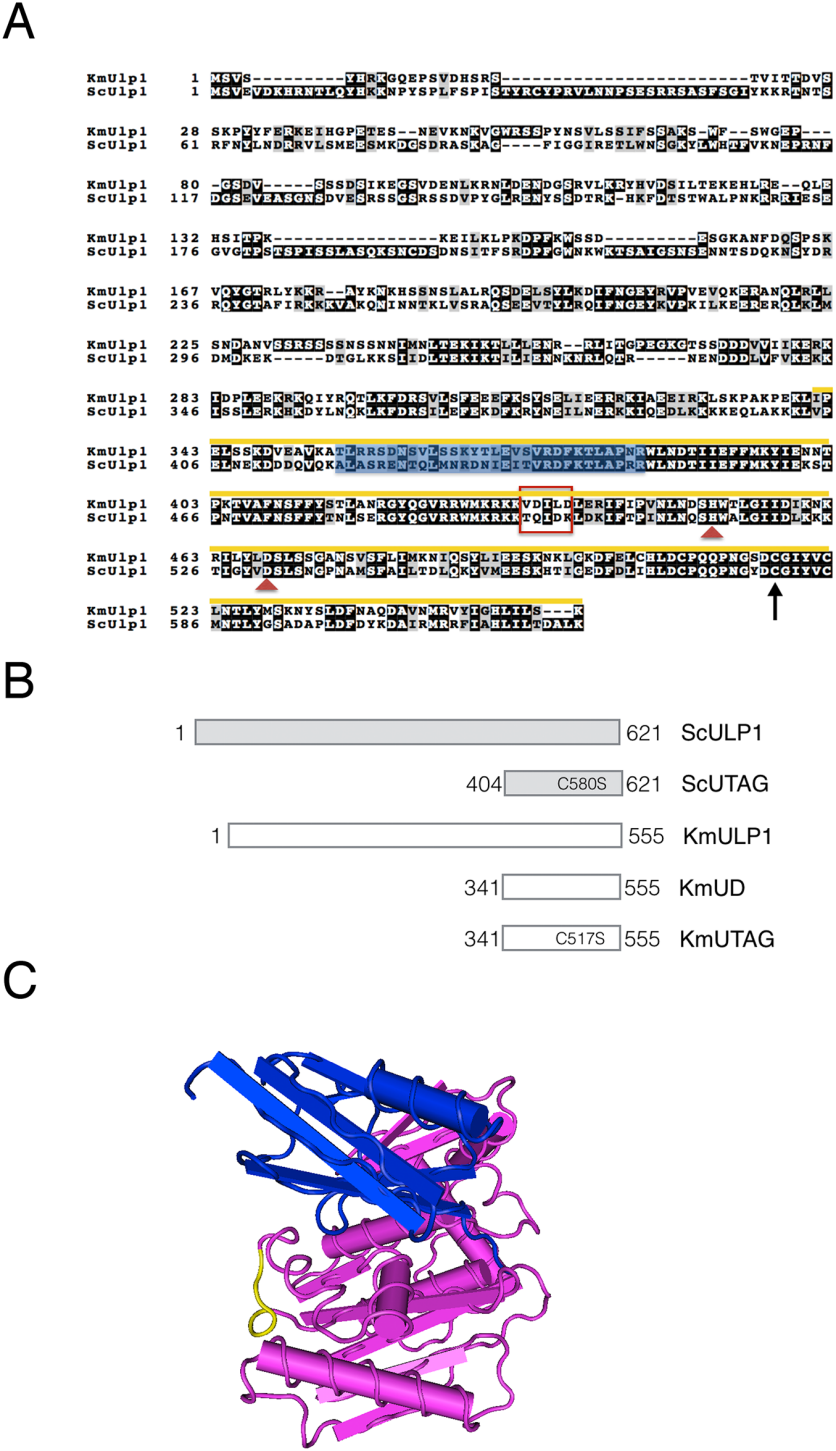
Alignment and features of the Ulp1 SUMO protease from *Saccaromyces cerevisiae* and *Kluyveromyces marxianus*. (**A)** Alignment of Ulp1 from *Kluyveromyces marxianus* (top) and *Saccharomyces cerevisiae* (bottom). The arrow indicates the catalytic cysteine at position C580 in ScUlp1 and C517 in kmUlp1. The yellow line indicates the region of the conserved catalytic UD domain of Ulp1 (NCBI #COG5160) characterized in this study and the blue highlight indicates a previously described SUMO-binding surface. A potential SIM (VDILD) that is present in KmULP1 but not ScULP1 is marked with a red box. (**B)** Schematic representation of SUMO proteases, truncations, and mutants studied in this work. (**C**) Three dimensional representation of the co-crystal structure of the catalytic domain of Ulp1 (Ulp1-UD, magenta) with yeast small ubiquitin-like modifier (SUMO/Smt3, blue). Indicated in yellow are the residues that correspond to the predicted SIM domain (VDILD) present in KmUlp1. The model was derived using MMDB database entry 13315.

We compared the sequences of budding yeast ScUlp1and KmUlp1 and noticed that, as expected, the catalytic UD domains of KmUlp1 and ScUlp1 showed a high degree of sequence conservation (63% identity and 80% similarity). Additionally, we noted that full-length KmUlp1 was 66 amino acids shorter than ScUlp1, which was consistent with a previous report on *K*. *marxianus* proteins. However, there is only a 3 amino acid difference in size between the UD domains of ScUlp1 and KmUlp1 (Fig 1A and 1B). One striking difference was the presence of a predicted SIM motif in the UD domain of KmUlp1 (VDILD) that is not found in the UD domain of ScUlp1 (GPS-SUMO [40]). This KmSIM-specific motif is not part of a previously described SUMO-binding surface of ScUlp1 (Fig 1A) [37]. We mapped the putative KmSIM onto the surface of the published co-crystal structure of ScUlp1 with budding yeast SUMO and found that it was surface exposed and accessible. If functional, this KmSIM may thus aid the interaction of KmUlp1 with SUMO and sumoylated proteins (Fig 1C). Based on this interesting observation we decided to focus on the conserved UD of KmUlp1 and to compare its SUMO-binding properties to those of ScUlp1 (see also Materials and methods).

### SUMO processing by KmUlp1

**Fig 2:**
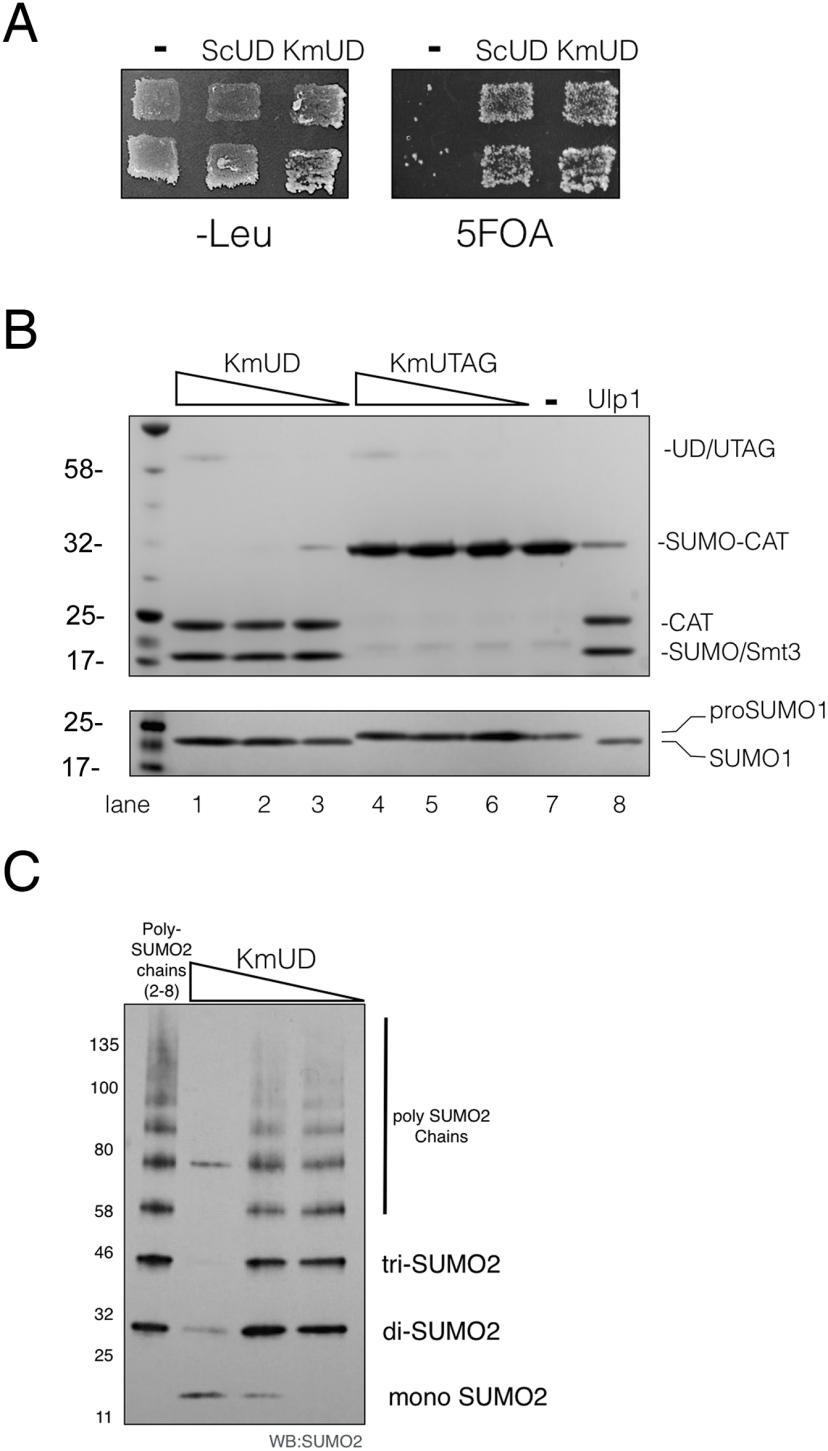
KmUlp1 is a SUMO protease. (**A**) Catalytically active UD fragments of ScUlp1 (ScUD) and KmUlp1 (KmUD) complement a *ulp1*::*HIS3* deletion in a yeast 5-FOA shuffle assay. An empty vector (-) does not support growth of *ulp1*::*HIS3* on 5-FOA and is included as a negative control. All transformed cell patches grow on media lacking 5-FOA (-Leu) (**B**) Purified catalytically active KmUD cleaves recombinant SUMO fusion protein (SUMO-CAT) (top gel) and pro-SUMO1 (bottom gel) *in vitro*. Cleaved recombinant proteins are labeled as CAT, SUMO and SUMO1, respectively. In contrast, the catalytically inactive KmUTAG carrying the C517S mutation is unable to process either protein. Commercially available Ulp1 is included as a positive control (2 units/Rx) and (-) indicates a negative control reaction without active or inactive SUMO proteases. Sloped triangle shapes indicate 10-fold serial dilutions of KmUD and KmUTAG (0.07μg each in the first 100μl reaction) used in this assay Proteins were resolved on an SDS-PAGE gel and stained using a Coomassie-dye. (**C**) KmUD can cleave wildtype human poly-SUMO2 chains that are linked via isopeptide bonds on lysine 11 *in vitro*. Each lane contains 0.6ug poly-SUMO2 (2–8). Cleavage reactions are shown below the sloped triangle shape and contain 10-fold serially diluted KmUD (0.14ug in the first reaction lane) as well as 0.6ug poly-SUMO2 (2–8). Proteins were resolved on an SDS-PAGE gel and western-blotted with an anti SUMO2/3 rabbit polyclonal primary antibody. Mono-, di, tri- and poly-SUMO2 adducts are as indicated. Numbers on the left indicate molecular weight markers.

ScUlp1 is an essential protein. Therefore, to assess whether KmUlp1 can substitute for the activity of ScUlp1 in budding yeast we cloned both catalytically active UD domains (Fig 1B). We found that both the ScUD and the KmUD, but not an empty vector, complemented an essential *ulp1*::*HIS3* deletion in a 5FOA shuffle assay at 30°C (Fig 2A). We reasoned that this ability of KmUD to complement the *ulp1*::*HIS3* deletion was due to its ability to process SUMO and sumoylated proteins. To confirm this prediction, we expressed fusion proteins of KmUD or of the catalytically inactive mutant, KmUTAG, with maltose-binding protein (MBP) in bacteria and tested their activity *in vitro*. Combining KmUD or commercially available Ulp1 in a reaction with the purified SUMO1 precursor (pro-SUMO) resulted in the formation of processed SUMO1 (Fig 2B top). In contrast, the KmUTAG failed to processes the SUMO precursor. Similarly, KmUD efficiently cleaved the Smt3 moiety off a purified Smt3-chloramphenicol acetyltransferase fusion protein (SUMO-CAT–Fig 2B bottom). SUMO-CAT was cut to completion by kmUD both at 30°C and 37°C. Finally, we tested the processing of physiological SUMO conjugates by KmUD (Fig 2C) Purified wildtype human poly-SUMO2 chains that are linked via isopeptide bonds on lysine 11 were incubated with varying concentrations of KmUD. Analysis of the poly-SUMO2 chains by western blotting using an anti SUMO2/3 antibody confirmed that SUMO chains were processed by KmUD in a concentration-dependent manner. These data suggest that, similar to ScUlp1, KmUlp1 can process SUMO precursors, SUMO fusion proteins, and SUMO conjugates.

### SUMO binding of KmUlp1

**Fig 3:**
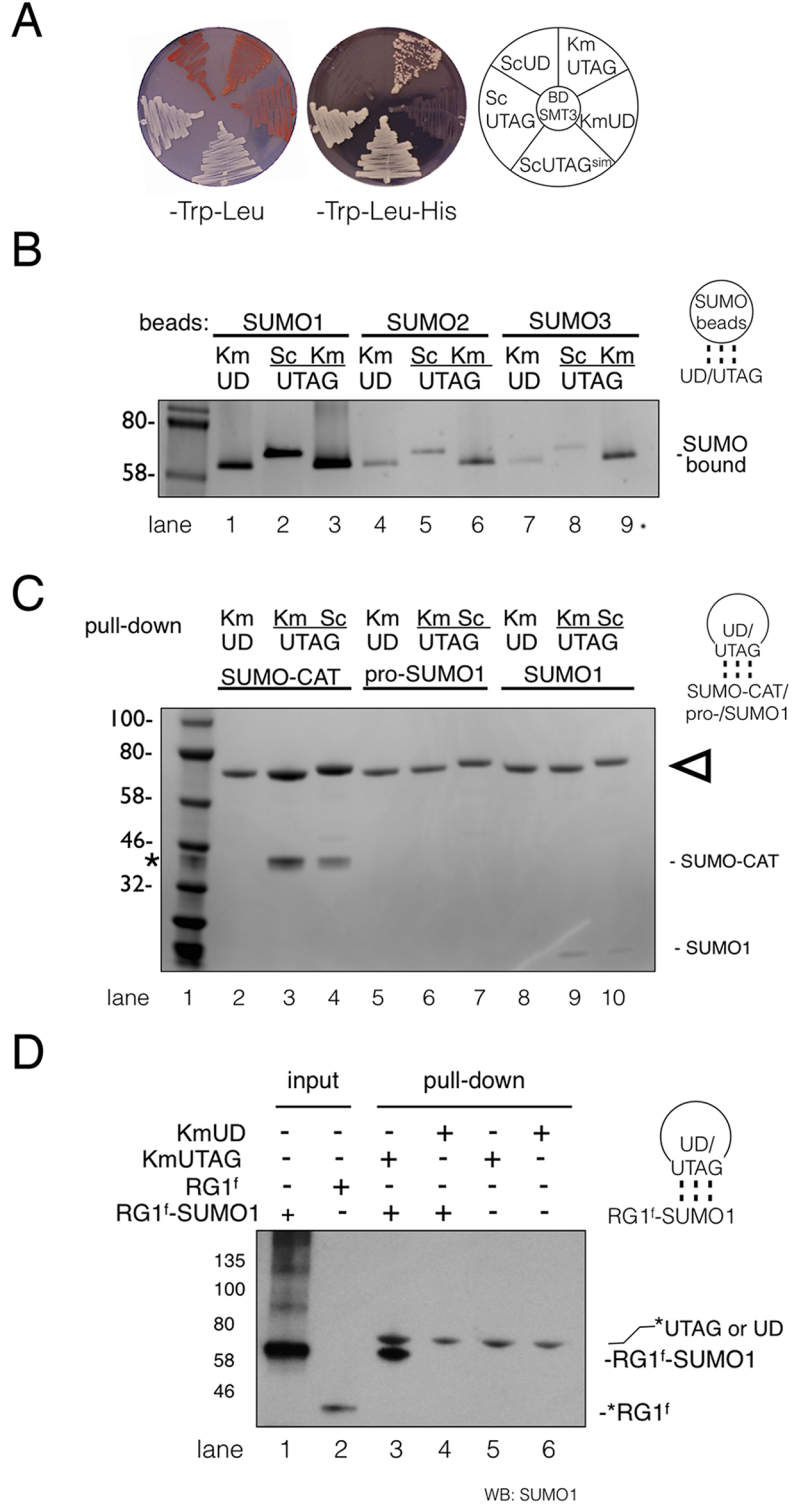
KmUTAG is a SUMO-binding protein. (**A**) Two-hybrid assay demonstrating that catalytically inactive KmUTAG, ScUTAG, and ScUTAG^SIM^ prey constructs (in *LEU2* marked pOAD) interact with a BD-SMT3/SUMO bait construct (in *TRP1* marked pOBD2) and activate a *HIS3* reporter construct as assayed on –Trp-Leu-His media. All transformed strains grow on –Trp -Leu media. Catalytically active KmUD and ScUD fail to activate the reporter. (**B**) Binding of recombinant KmUD, ScUTAG and KmUTAG to SUMO1, SUMO2, and SUMO3-conjugated agarose beads. Proteins were bound to the indicated beads in the presence of the reducing agent TCEP in SUMO Protease Buffer (SPB: See Materials and methods). After 3 washes proteins were eluted, resolved on SDS-PAGE gels, and stained using a Coomassie G-250 stain. Lane 1 in all figures corresponds to the protein ladder with molecular weights indicated in kDa. (**C**) Binding of recombinant MBP (maltose binding protein) fusions of KmUD, ScUTAG and KmUTAG to a soluble SUMO fusion protein (SUMO-CAT), SUMO1, and pro-SUMO. After incubation in SPB with TCEP, protein complexes were pulled down using amylose resin. Proteins were resolved on SDS-PAGE gels, and stained as above. Arrows indicate UD and UTAG proteins. Also indicated are SUMO-CAT and SUMO1. 6μg of each protein was used for binding assay and half of the binding reactions were run on the gel (compare also Fig 2B). (*) indicates 1μg of SUMO-CAT that was included as a loading control in the lane with the ladder. SUMO1 and proSUMO1 inputs are show in the S2 Fig (**D**) KmUTAG binds an *in vitro*–sumoylated fragment of RanGAP1 (RG1^f^). RG1^f^ (lane 2) was sumoylated *in vitro* with SUMO1 to produce RG1^f^ –SUMO1 (lane 1) (see Materials and methods). RG1^f^ –SUMO1 was incubated with KmUTAG or KmUD as indicated and pulled down using amylose resin. Proteins were resolved on SDS-PAGE gels and western blotted with an antibody to SUMO1. In this western blot the anti SUMO1 antibody cross-reacted with KmUTAG, KmUD and RG1^f^ as indicated on the blot (*). Note that RG1^f^ –SUMO1 was only pulled down with KmUTAG (lane 3), but not KmUD (lane 4). Sumoylated RG1 is cleaved by catalytically active KmUD1 (lane 4) and is then removed with the washes before elution (see Materials and methods). Graphic representations to the right of B,C,D indicate whether SUMO or a protease domain was immobilized on the beads (circles) and which protein was bound.

In our sequence alignment the catalytic cysteine (C580) of ScUlp1 corresponds to C517 of KmUlp1 (Fig 1A and 1B). We previously showed that when cysteine (C580) of ScUlp1 UD was changed to a serine, the mutant recombinant protein (ScUTAG) was rendered catalytically inactive but interacted avidly with sumoylated proteins [19]. To study the SUMO-binding of KmUlp1 we introduced the corresponding C517S mutation into the KmUD to form a SUMO-trapping KmUTAG protein. First, we assayed the ability of KmUTAG to interact with SUMO in a two-hybrid assay (Fig 3A). In this assay we found that Gal4-activation domain (AD) fusions of both ScUTAG and KmUTAG interact with the Gal4-DNA binding domain (BD) fusion of yeast SUMO/Smt3. In contrast, both catalytically active ScUD and KmUD did not activate the *HIS3* reporter gene and failed to support growth on media lacking histidine (Fig 3A). We then investigated the binding of recombinant KmUD, ScUTAG and KmUTAG to beads coated with non-cleavable SUMO1, 2 or 3. Bound proteins were eluted, separated by SDS-PAGE, and visualized using a Coomassie G250 stain. In this *in vitro* binding assay, KmUD, KmUTAG, and ScUTAG interact avidly with SUMO1. All proteins also bind to SUMO2 and SUMO3 binding beads, albeit less efficiently (Fig 3B). SUMO beads used in this study contained equivalent or very similar amounts of SUMO1, SUMO2, or SUMO3 (4.9–5.0 mg/ml or ~0.45μM respectively). However, SUMO1 is most similar to Smt3 (~50%), and this may explain why the yeast-derived KmUD, KmUTAG, and ScUTAG are preferentially enriched on SUMO1 beads. This is consistent with our previous finding that the C580S containing ScUTAG is a SUMO-trapping protein [19]. Next, we investigated the binding of KmUD, KmUTAG, and ScUTAG to a soluble, recombinant SUMO-CAT fusion protein, unbound SUMO1, and the pro-SUMO1 precursor (Fig 3C). As we show above, KmUD is catalytically active (Fig 2B) and did not precipitate SUMO-CAT, pro-SUMO1 or SUMO1 (Fig 3C lanes 2, 5, 8). In contrast, KmUTAG and ScUTAG efficiently precipitated the SUMO-CAT fusion protein (Fig 3C lanes 3 and 4). We were also able to detect a small amount of SUMO1 (Fig 3C lanes 9 and 10) but no pro-SUMO1 (Fig 3C lanes 6 and 7). This may indicate that KmUTAG and ScUTAG preferentially remain bound to proteins that have been modified with SUMO. Finally, we also investigated the ability of KmUTAG to interact with an *in vitro*–sumoylated RanGAP1 fragment (RG1^f^). To assess binding, the SUMO1-modified RG1^f^ was combined with KmUTAG or KmUD (Fig 3D). SUMO1-modified RG1^f^ efficiently precipitated with KmUTAG, but not KmUD, as shown by western blotting with an anti-SUMO1 antibody. In summary, these experiments suggest that SmUTAG and KmUTAG are pan-SUMO binding proteins with a preference for SUMO conjugates.

### SUMO-binding under stress

**Fig 4:**
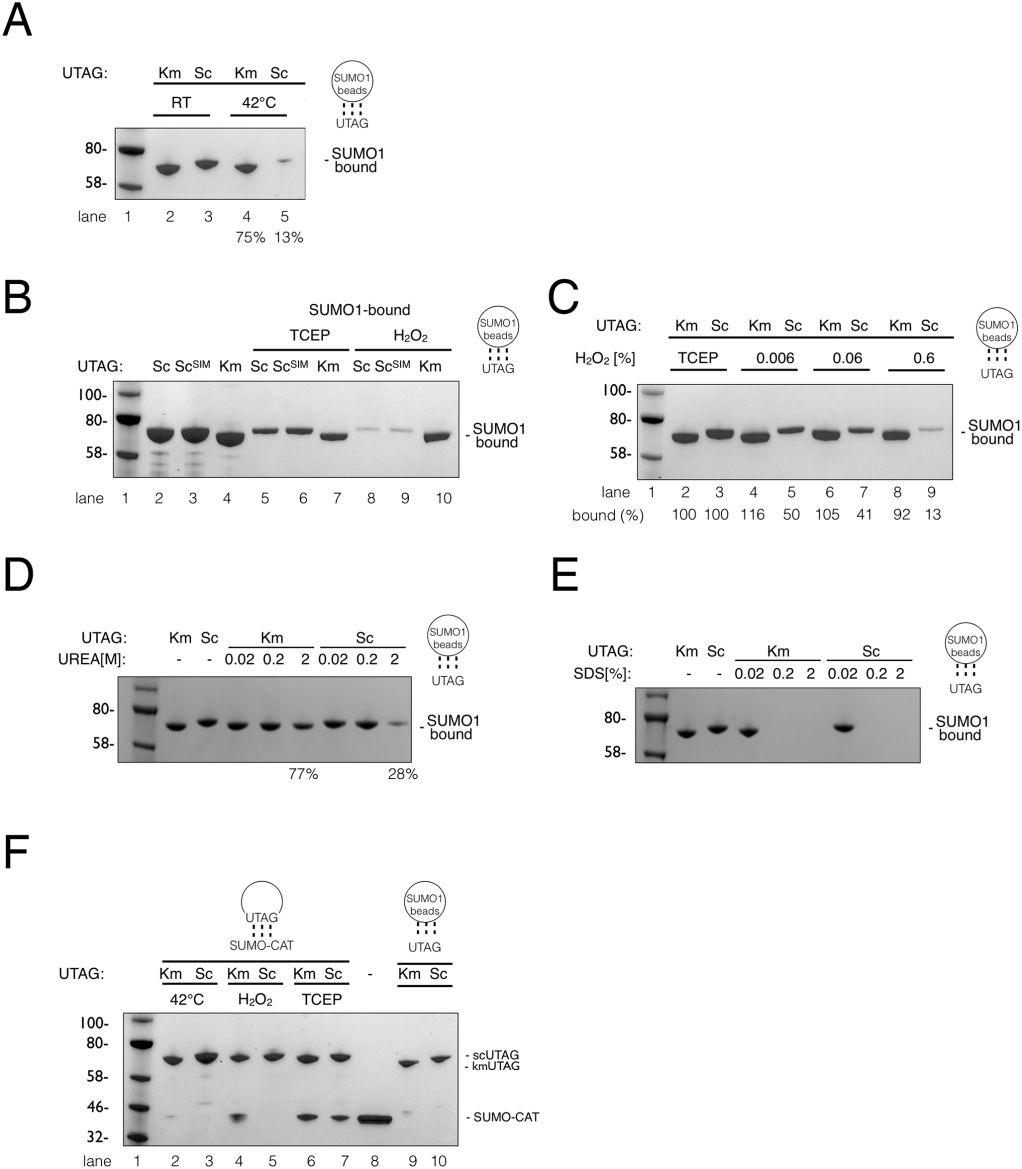
KmUTAG SUMO-binding under stress conditions. (**A**) Analysis of SUMO-binding under heat stress. Recombinant KmUTAG or ScUTAGs were incubated with SUMO1 beads in the presence or absence of the indicated treatments: with TCEP at room temperature (lanes 2 and 3) and at 42°C (lanes 4 and 5). Subsequently, SUMO beads with bound UTAG proteins were visualized and quantitated using a BioRad imager and BioRad Image Lab software. (**B**) Three recombinant UTAG proteins, ScUTAG, ScUTAG containing the putative KmSIM and KmUTAG (lanes 2–4), were incubated with SUMO1-conjugated agarose beads in the presence of a reducing agent TCEP [5mM] or 1% hydrogen peroxide (H_2_O_2_). SUMO1-bound UTAG proteins in TCEP-containing buffer (lanes 5–7) or in peroxide-containing buffer (lanes 8–10) were eluted and visualized as above. Reduced binding of both ScUTAG proteins to SUMO1 beads was quantitated as above. (**C**) Analysis of SUMO-binding in the presence of various concentrations of hydrogen peroxide (H_2_O_2_). KmUTAG or ScUTAG proteins were incubated with SUMO1 beads in the presence or absence of the indicated concentrations of hydrogen peroxide (lanes 4–9), without hydrogen peroxide (lanes 2 and 3). Subsequently, eluted UTAG proteins were visualized and quantitated as above. (**D & E**) Analysis of SUMO-binding in the presence of various concentrations of urea and SDS. Recombinant KmUTAG or ScUTAGs in the presence of urea (0.02 M, 0.2 M, 2.0 M) or SDS (0.02%, 0.2%, 2.0%) as indicated. All binding reactions were performed in the presence of 5mM TCEP. SUMO1 binding reactions without urea and SDS are included as controls (**F**) Binding of UTAG to a SUMO-fusion protein. Recombinant HIS6-SUMO-CAT (a linear fusion protein of a HIS6 affinity tag, Smt3/SUMO, and chloramphenicol acetyl transferase) was incubated with KmUTAG or ScUTAG (these UTAGs were produced as fusions with the maltose binding protein MBP). Individual reactions were incubated as follows: at 42°C (lanes 2 and 3), in the presence of 0.6% hydrogen peroxide (H_2_O_2_) (lanes 4 and 5), or in the presence of 5mM TCEP (lanes 6 and 7). After incubation at the stated conditions, amylose beads were used to pull down the UTAG and associated SUMO-CAT. UTAG and co-purifying proteins were eluted with 2x SDS-PAGE sample buffer, visualized and quantitated as detailed above. Also shown for comparison and as controls are samples of recombinant SUMO-CAT (lane 8) and SUMO1 purified KmUTAG (lane 9) and ScUTAG (lane 10). Graphic representation to the right of A-F indicate which proteins were linked to SUMO beads or amylose resin and which proteins were pulled down.

Considering that *K*. *marxianus* is a thermotolerant yeast, we compared the SUMO-binding ability of KmUTAG to ScUTAG under conditions that impede proper protein folding and protein/protein interactions. Initially, we compared the binding of purified KmUTAG and ScUTAG proteins to SUMO1 beads at 25°C (RT) and 42°C. *K*. *marxianus* is known to withstand temperatures up to 49°C and this observation was reflected in the binding to SUMO1 beads. Both UTAGs showed robust binding to SUMO1 beads at RT, but at 42°C ScUTAG binding was greatly reduced (87% reduction of ScUTAG) while the interaction between KmUTAG and SUMO1 was much less affected (25% reduction) (Compare Fig 4A lanes 4 and 5). We reasoned that the recombinant KmUTAG protein is more stable at 42°C and so we also attempted our SUMO1-binding reaction in the presence of hydrogen peroxide (H_2_O_2_), a compound known to induce oxidative damage of proteins and the oxidation of the catalytic cysteine in SUMO proteases. Intriguingly, we observed that KmUTAG is able to bind SUMO1 beads even in the presence of 0.6% H_2_O_2_ while ScUTAG binding under these conditions is reduced to 17% (Fig 4B compare lanes 8 and 10). A ScUTAG protein that was mutated to express the proposed SIM residues of KmUTAG did not reveal a significant enhancement of SUMO1-binding in the presence of peroxide (Fig 4B compare lanes 8 and 9). Further analysis showed that the SUMO-binding activity of ScUTAG was extremely sensitive to peroxide with even minute levels of peroxide (0.006%) reducing the ability to bind SUMO1 by 50% (Fig 4C). Since the catalytic cysteine of ScUTAG and KmUTAG is replaced by a serine, these data suggest that oxidation of another residue or possibly oxidative damage to multiple residues impedes SUMO binding.

The apparent stability of KmUTAG prompted us to further test its SUMO-binding resilience. First we tested the ability of the chaotrophic protein denaturant urea and the anionic detergent SDS to impede binding of KmUTAG and ScUTAG to SUMO1 beads. Both KmUTAG and ScUTAG bound SUMO1 beads in the presence of up to 0.2M urea. Binding of KmUTAG was reduced by 23% in 2M urea while ScUTAG binding was reduced by 82% (Fig 4D). This data suggests that KmUTAG is overall less prone to denaturation than ScUTAG. In contrast, both KmUTAG and ScUTAG were sensitive to 0.2% SDS, as all binding to SUMO1 beads in the presence of this detergent was prevented (Fig 4E). Similarly, both KmUTAG and ScUTAG failed to bind to SUMO1 in the presence of deoxycholate, an ionic detergent known to disrupt protein/protein interactions, and N-ethylmaleimide, a compound that forms stable, covalent thioether bonds with sulfhydryls. The latter, NEM-mediated inhibition of SUMO1-binding warrants further investigations because both KmUTAG and ScUTAG harbor serine replacement mutations of their catalytic cysteines, suggesting that additional NEM-modified cysteines interfere with SUMO-binding. As controls, incubation in the presence of 1% ethanol or 1mM PMSF did not affect SUMO1-binding.

Finally, we also tested how binding of KmUTAG and ScUTAG to the soluble SUMO-CAT fusion protein is affected by various stressors. Purified SUMO-CAT was combined either with KmUTAG or ScUTAG. After incubation in the presence or absence of H_2_O_2_ or at 42°, MBP fusions of KmUTAG or ScUTAG were precipitated using amylose resin. Co-purifying proteins were eluted from washed beads, separated by SDS PAGE and stained with a Coomassie dye. As was seen with the SUMO1 beads, KmUTAG but not ScUTAG was able to bind SUMO-CAT in the presence of H_2_O_2_ (Fig 4F compare lanes 4 and 5). At 42°C, binding of either UTAG to SUMO-CAT was greatly reduced or absent (Fig 4F lanes 2 and 3). One possible explanation for this observation is that the SUMO-CAT substrate denatures at this temperature. We also wanted to assess the contributions of the putative SIM in KmUTAG on the stress tolerance. Therefore, we replaced the core SIM in kmUTAG (VDIL) with the equivalent sequence (TQID) of ScUlp1 to form kmUTAG^sim^. Unexpectedly, the kmUTAG^sim^ mutant failed to bind SUMO1 beads even at ambient temperature (see S1 Fig) and potential reasons for this unexpected observation are discussed below. Nevertheless, these data reveal that SUMO-binding features of *K*. *marxianus* Ulp1 reside in its catalytic domain and have evolved with exceptional resilience to oxidation and protein-misfolding stressors.

### SUMO-binding kinetics of KmUTAG

**Fig 5:**
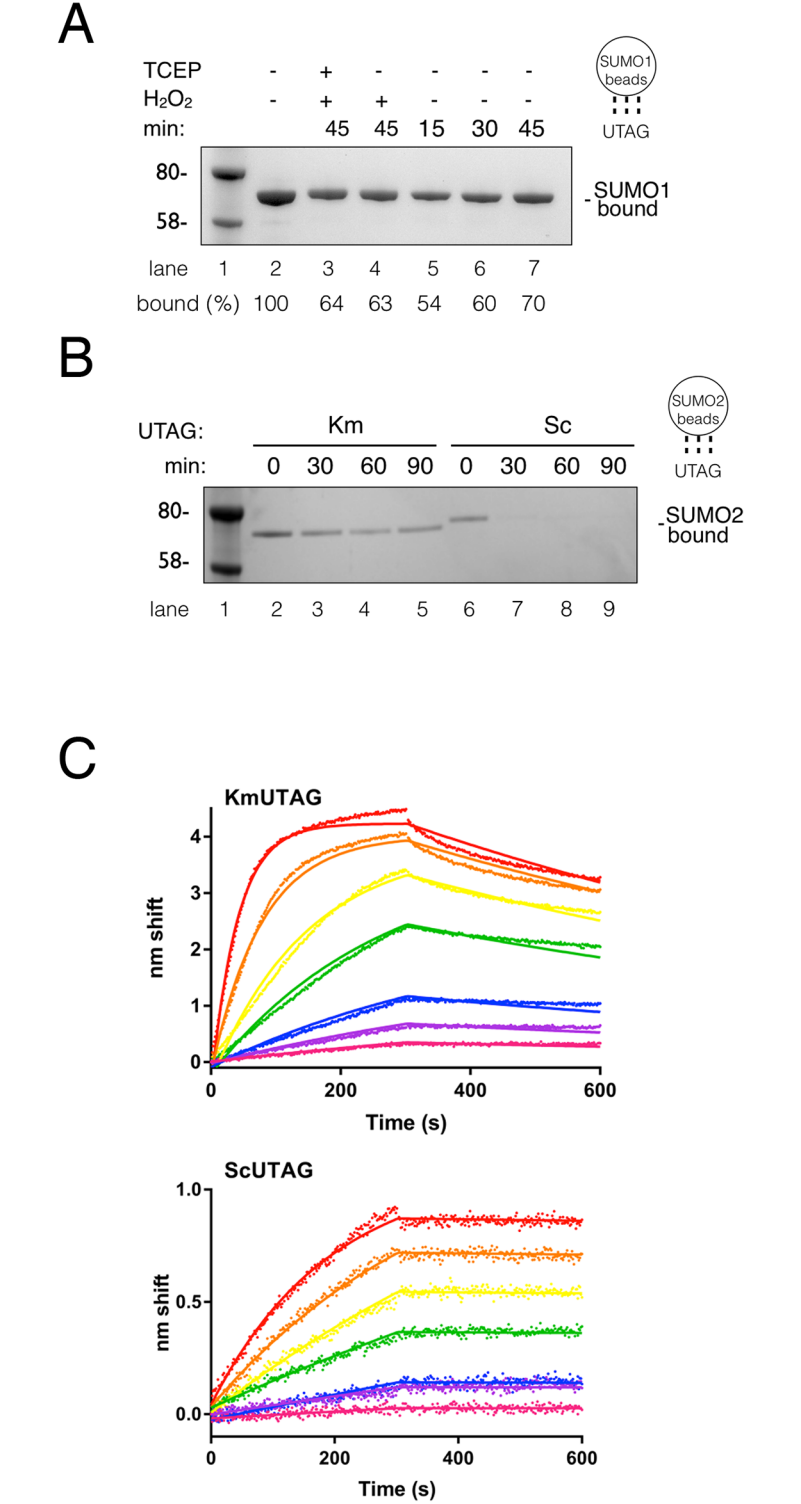
Analysis of binding kinetics of the KmUTAG protein to SUMO. (**A**) Lanes 2–7: Binding of KmUTAG to SUMO1 beads over time (min) or in the presence or absence of hydrogen peroxide (H_2_O_2_) as indicated. Lane 2: total protein in each binding reaction. (**B**) Retention of bound KmUTAG (Km) or ScUTAG (Sc) on SUMO2 beads over time (min). (**C**) Sensorgrams for KmUTAG (top) and ScUTAG (bottom) binding to biotinylated SUMO1. Analyte UTAG concentrations were 250 nM (red), 125 nM (orange), 63 nM (yellow), 31 nM (green), 16 nM (blue), 8 nM (purple) and 4 nM (magenta). Raw data (points) are shown with fits (solid lines) to a global one-state binding model. Association was 0–300 s. Dissociation was 300–600 s. Graphic representations to the right of A and B indicate that SUMO1 or 2 was immobilized on the beads (circles) and that KmUTAG was precipitated.

We sought to investigate the SUMO-binding kinetics of KmUTAG. First, we tested KmUTAG’s association with SUMO1 beads over time. We found that binding was rapid and that within 15 minutes of incubation 50% of the protein was bound to SUMO1 beads. A maximum of 70% was achieved after 45 minutes of incubation (Fig 5A). In the presence of peroxide about 60% of KmUTAG was bound to SUMO1 beads after 45 minutes (Fig 4A lanes 3 and 4). Second, we investigated how KmUTAG was retained on SUMO beads. For this experiment KmUTAG or ScUTAG was bound to SUMO2 beads and after initial binding for 45 minutes, the beads were washed for the indicated times. The results show that KmUTAG but not ScUTAG is retained for 90 minutes during the extended washes. Little or no ScUTAG is retained after 30 minutes. Binding to SUMO1 beads also showed an avid binding of KmUTAG even after prolonged washes. This association and retention over time was analyzed quantitatively using biolayer interferometry, an optical biosensing technique similar to surface plasmon resonance (Fig 5C). We analyzed the binding of KmUTAG and ScUTAG to immobilized biotinylated SUMO1 at 25°C (Fig 5C, KmUTAG (top) and ScUTAG (bottom)). When fit to a single-state binding model, K_D_s were 8.7 nM for KmUTAG and 2.0 nM for ScUTAG. KmUTAG exhibited faster binding (kon = 9.6 x 10^4^ M^-1^s^-1^ vs. 2.1 x 10^4^ M^-1^s^-1^ for ScUTAG) but also faster dissociation (k_off_ = 8.4 x 10^−4^ s^-1^ vs 4.2 x 10^−5^ s^-1^). While there is a secondary component apparent in KmUTAG binding, elimination of the top two analyte traces from analysis changed K_D_ only modestly (8.7 nM). KmUTAG did not fit a two-state parallel model, indicating that the secondary component, if biologically relevant, is complex.

### *In vitro* and *in vivo* SUMO-targeting of KmUTAG

**Fig 6:**
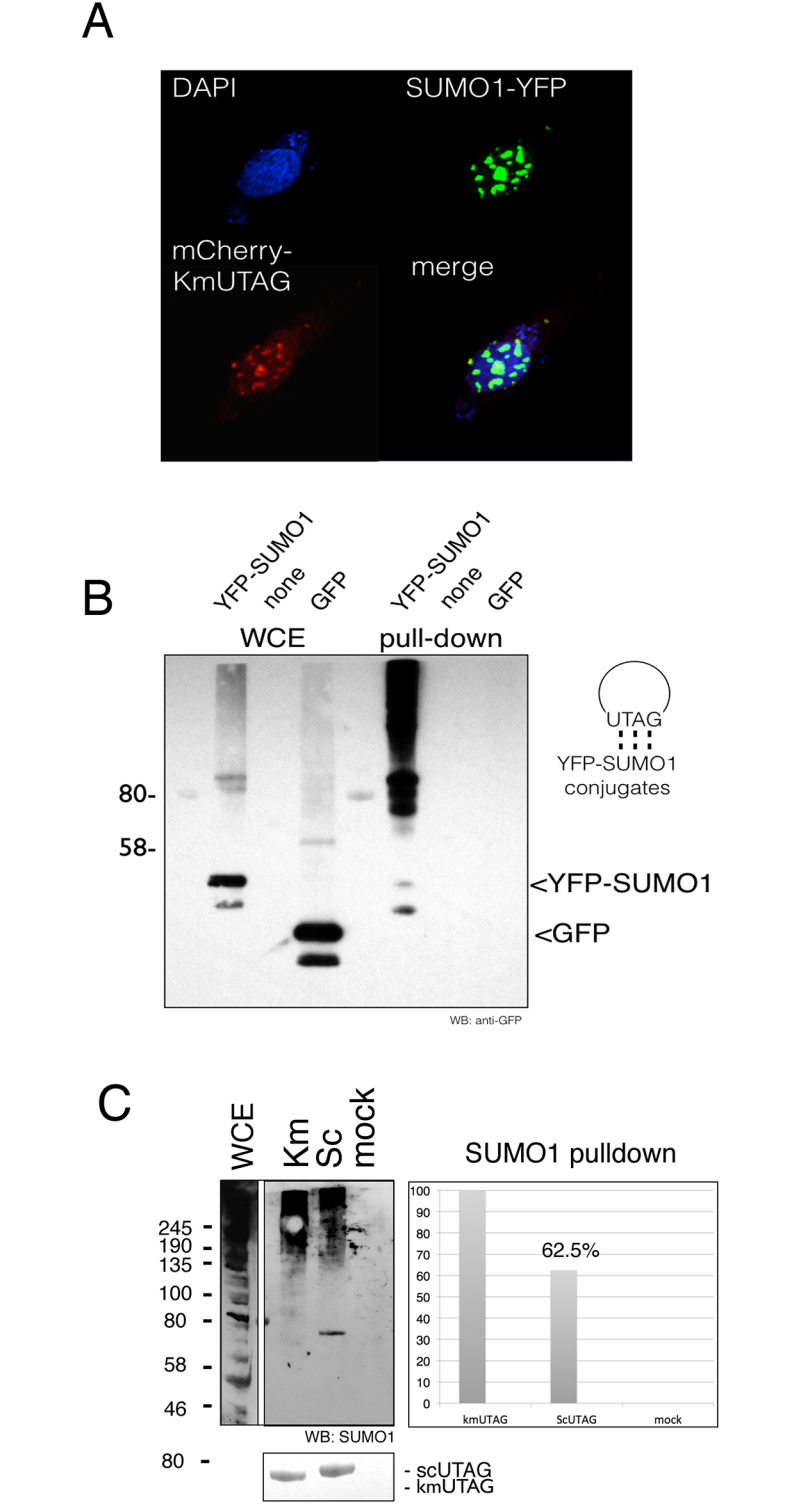
UTAG expression in mammalian cells. (**A**) UTAG localization to sites of sumoylation in mammalian cells. mCherry-tagged KmUTAG and pEYFP-SUMO1 were transfected into mammalian cells that were grown on cover slips. Cells were then fixed, stained with DAPI, and visualized using a confocal microscope. An overlay of DAPI, mCherry, and YFP images is shown in the lower right corner (merge). Expression of kmUTAG did not alter the distribution or localization of YFP-SUMO (**B**) Using UTAG to pulldown SUMO-modified proteins from mammalian cell extracts. Whole cell extracts (WCE) of 786–0 renal carcinoma cells transfected with YFP-SUMO1, control GFP or untransfected (none). Pulldowns using recombinant KmUTAG on the right. Note high molecular weight YFP-SUMO1 conjugates in the pulldown of YFP-SUMO1. The graphic representation to the right indicates that KmUTAG was immobilized on amylose beads (circle) and that YFP-SUMO conjugates were pulled down. (C) 5ug KmUTAG, SCUTAG or no protein (mock) were added to 100ul diluted cell extracts (see Materials & methods) and nutated in the presence of nickel beads for 1 hour at 25°C. Eluted proteins were run on SDS-PAGE, western blotted with anti SUMO1 antibody, and quantitated using a c-DIGIT scanner (Li-Cor Biosciences). 20% of input whole cell extract (WCE) probed with the anti SUMO1 antibody is shown for comparison.

When expressed in living cells, catalytically inactive GFP-tagged SUMO proteases are enriched at sites of SUMO modification. For example, in living yeast cells GFP-tagged *Ulp1*^*(C580S)*^ localizes to septins after nocodazole-induced G_2_/M arrest, and in mammalian cells *SENP1(C603S)* mutant localizes to PML nuclear bodies and domains of the HDAC4 protein [19,41]. Therefore, we were curious to determine whether KmUTAG could be used to detect sumoylated proteins in cultured mammalian cells. To this end we created a codon-optimized mCherry-tagged kmUTAG construct that was transfected in 786–0 renal carcinoma cells together with YFP-tagged SUMO1. Transfected cells were grown on cover slips for 24 hours in DMEM media before fixation and imaging using a Nikon Confocal microscope. Z-sections of representative cells were collected using the appropriate filter sets (FITC, TRITC, UV) to differentiate between YFP (SUMO1), mCherry (kmUTAG), and DAPI (chromatin) signals. As previously reported, the majority of YFP-SUMO1 localized to the nucleus and was enriched in numerous nuclear (PML) bodies (Boudreau 2012). In all cells that displayed a clearly discernible SUMO1 signal, mCherry-tagged kmUTAG co-localized with SUMO1, suggesting that the pan-SUMO binding KmUTAG associates with sumoylated proteins in living cells (Fig 6A). We also used KmUTAG to purify sumoylated proteins from extracts of 786–0 cells. Extracts were prepared from untransfected 786–0 cells, from GFP-only transfected cells, and from YFP-SUMO1-tranfected cells. Cleared extracts were then incubated with 6 μg of recombinant KmUTAG and Talon beads were used for pulldowns (see Materials and methods). After western blotting with an anti-GFP antibody, both GFP and YFP-SUMO1 were detected in the whole cell extract (WCE). In contrast, only YFP-SUMO1 was detected in the KmUTAG pulldowns, demonstrating the specificity of this pan-SUMO trapping protein (Fig 6B). Similarly, we compared the ability of recombinant KmUTAG and ScUTAG to pulldown SUMO1 conjugates from peroxide-treated human prostate cancer (PC-3) cells (Fig 6C). For this comparison a mock-pulldown without either SUMO-trapping protein was includes as a control. Levels of SUMO1 in the pull-downs and the control were then analyzed using quantitative western blotting. Our results suggest that under these conditions kmUTAG is ~40% more effective in pulling down SUMO1 conjugates. Therefore, we predict that KmUTAG may be a useful reagent for the detection of SUMO and sumoylated proteins in many different types of eukaryotic cells. Additionally, these data show for the first time the application of a stress and thermo-tolerant SUMO-trapping Ulp1 SUMO protease mutants such as KmUTAG for the specific detection and purification of SUMO-modified proteins.

## Discussion

DMKU 3–1042 is a particularly stress-resistant strain of the thermotolerant hemiascomycetous yeast *K*. *marxianus*, making it an attractive organism for biofuel and recombinant protein production. The stress tolerance of *K*. *marxianus* is positively correlated to the abundance of transcripts for ribosome assembly, translation, transcription, DNA repair, and scavenging reactive oxygen species (ROS) [42].

Additionally, the reduction in primary sequence length of *K*. *marxianus* proteins has been hypothesized to be an important contributing factor to their superior thermotolerance and unfoldability. A recent study took advantage of these stress-tolerant features to clone a suite of thermostable autophagy core proteins for the elucidation of molecular details of autophagy [39]. Similar to autophagy-related proteins, components of the SUMO system are also conserved in *K marxianus*. Studying SUMO pathway proteins expressed in this thermotolerant yeast may, therefore, yield useful molecular details about the transient interactions with SUMO and its substrates, especially under stress conditions.

Our ultimate goal was to compare and contrast determinants of SUMO targeting, binding, and processing in Ulp1 SUMO protease of *K*. *marxianus* and *S*. *cerevisiae*. Next to Ulp1 we were also able to identify putative orthologs of Ulp2, Smt3, Siz1, Mms21, Ubc9, Aos1, Uba2 and also Slx5 and Slx8 (see S1 Table and https://www.ncbi.nlm.nih.gov/genome/10898). We failed to detect a match for budding yeast Siz2 raising the possibility that, similar to *S*. *pombe*, *K*. *marxianus* may only express two SUMO E3 ligases, Siz1 and Mms21/Nse2. We also found that SUMO pathway components, except KmUlp2, were generally shorter than their budding yeast orthologs, and this is consistent with a previously published comprehensive bioinformatics analysis of *K*. *marxianus* and *S*. *pombe* proteins [39]. We observed the biggest size difference between KmUlp1 and ScUlp1 (66 amino acids) and Km and Sc Siz1 (99 amino acids). Using the *Foldindex* webs-tool, we generated the predicted unfoldability scores for all SUMO pathway components [43]. We found that, as expected, Km SUMO pathway components were general less disordered (see the S1 Table). Consistent with its predicted thermotolerance, the UD domain of KmUlp1 (+0.136) showed a dramatic reduction in disordered segments when compared to ScUlp1 (+0.09) even though both protein domains only differed by 3 amino acid residues (215 vs 218).

One prediction that we investigated is that growth at elevated temperatures may necessitate additional features of KmUlp1 that facilitate its productive interaction with SUMO, SUMO conjugates and SUMO-chains under conditions that are unfavorable for protein interactions. Focusing on the catalytic domain of KmUlp1 and ScUlp1, we noted a surface-exposed putative SIM in the KmUD domain. This SIM (VDILD) is of the h-X-h-h type followed by a negative charge (D) and was defined using the high threshold on GPS-SUMO [40]. This putative SIM is not conserved in the ScUD domain (TQIDK). This is interesting because SIMs have not been reported to reside in the UD domains of SUMO proteases. However, upon further investigation we found the identical sequence motif in putative orthologs of Ulp1 in *K*. *lactis (XP_455581*.*1) and K*. *dobzhanskii (CDO95827*.*1)*.

To study the SUMO-binding properties of KmUlp1 we first showed that, analogous to ScUlp1, KmUlp1 binds and cleaves a variety of SUMO isoforms and SUMO conjugates. Next, we used a SUMO-trapping mutant of KmUlp1, KmUTAG, to investigate SUMO binding in the presence of proteotoxic stress. Our analysis indicates that SUMO-trapping by KmUTAG is considerably more stress-tolerant than the previously described SUMO-trapping by ScUTAG [19]. For example, SUMO-trapping by KmUTAG is little or not at all affected by elevated temperature (42°C), the presence of oxidative stress (0.6% hydrogen peroxide), or the presence of a strong protein denaturant (2M urea). Under these conditions, SUMO-trapping by ScUTAG is greatly reduced or absent. However, both KmUTAG and ScUTAG fail to bind SUMO in the presence of SDS (0.2%), NEM (5mM), and deoxycholate (0.5%). It should also be noted that the ability of KmUTAG to trap SUMO-modified proteins will depend on the solubility of individual SUMO-modified proteins and whether SUMO is properly oriented and accessible.

We tested the putative SIM of KmUTAG detailed above. First, we replaced the IDKLD sequence in ScUTAG with VDILD. However, ScUTAG with the VDILD motif remained sensitive to peroxide treatment and failed to bind to SUMO1 beads (Fig 4B). Next, we replaced VDIL in KmUTAG with the TQID sequence and this SIM replacement mutant failed to bind SUMO1 beads even at ambient temperatures. One possible interpretation of this observation is that KmUlp1, but not ScUlp1, may rely on the VDILD SIM motif to bind SUMO-modified proteins. However, at this point we cannot exclude the likely possibility that replacement of the SIM alters the folding of the KmUD domain and prevents SUMO binding. We also attempted to replace the putative VDILD SIM with alanines but the purified protein precipitated after cryostorage. Confirmation of this potential SIM in KmUlp1 and its functional relevance awaits further analyses. We continue to test our model that the putative SIM and the SBS of KmUD cooperate to bind poly-sumoylated proteins or SUMO chains

We reasoned that additional difference could be apparent from the kinetic analysis of both UTAG proteins. BLI analysis shows that both KmUTAG and ScUTAG bind SUMO1 with high affinity, exhibiting rapid-on, slow-off binding from which we can conclude that both analytes are kinetically very similar, at least at 25°C. The anomalous difference in signal amplitudes, much higher for any given concentration of KmUTAG, may result from differences in quantitation of the UTAGs or some other unknown factor, e.g. shape (compare Fig 5C traces in top and bottom graphs). They may, however, be reflective of some biologically relevant difference of KmUTAG such as oligomerization. We hope to characterize any such differences in a future study.

Another question concerns the peroxide-resistance of KmUTAG. It has been reported that ROS inactivate Ulp-type SUMO proteases either through a reversible intermolecular dimerization or the irreversible oxidation of the catalytic cysteines [28]. KmUTAG relies on the same catalytic residues as the peroxide-sensitive ScUTAG. How KmUTAG achieves its peroxide resistance is, therefore, not immediately clear. One interesting possibility is that an additional surface-exposed cysteine residue (C504) that is present in the UD-domain of KmUlp1 contributes to the formation of a protective, reversible intermolecular disulfide bond under conditions of oxidative stress. Similarly, KmUlp1 is missing a lysine (K602 in ScUlp1) that has been implicated in preventing the reduction of an overoxidized cysteine in ScUlp1. However, there is currently no experimental evidence to support either of these hypotheses.

In summary, our observations suggest that KmUlp1 has evolved to not only withstand denaturation but to functionally interact with SUMO even under extreme conditions. Conceivably, since *K*. *marxianus* can grow at temperatures above 49°C and is resistant to proteotoxic stress, KmUlp1 had to evolve to maintain its SUMO targeting, binding, and processing activity under adverse conditions [44]. This is particularly interesting since heat-shock and oxidative stress are known to inactivate SUMO proteases and lead to the accumulation of SUMO conjugates and SUMO chains in cultured mammalian cells [28,45]. Recent work has shown that SUMO and SUMO chains accumulate in the nucleus at sites of active transcription and are hypothesized to protect protein complexes during proteotoxic stress [23,25,46]. As cells recover from heat shock, SUMO chains are depolymerized, presumably as SUMO protease activity is recovered and SUMO-targeted ubiquitin ligases (STUbLs) antagonize the formation of SUMO chains and proteins modified with SUMO [29,47].

Due to its pan-SUMO binding properties and its ability to bind SUMO in the presence of proteotoxic reagents, KmUTAG may represent a useful reagent for the detection and purification of SUMO conjugates and SUMO chains that form under stress. For example, we were able to show that recombinant kmUTAG is more effective in the purification of SUMO1 conjugates from peroxide-treated PC-3 cells than ScUTAG (Fig 6C). Albeit, identification of these SUMO conjugates is beyond the scope of this paper our findings provide evidence that this strategy to precipitate and visualize sumoylated proteins may be aided by the stress-tolerant Km version of this SUMO-trapping protein fragment. Ultimately, we plan to transfect the stress-tolerant KmUTAG expression construct into mammalian tissue culture cells to trap sumoylated proteins as they accumulate during heat or oxidative stress. The KmUTAG may not be affected by these treatments (e.g. denature or become inactivated) and bound SUMO-modified proteins can then be identified using mass spectrometry or specific antibodies. Furthermore, since KmUTAG is a single–chain SUMO-trapping protein, we are using a cell-penetrating CPP-adaptor system to deliver and release these small stress-tolerant SUMO-trapping proteins into the cytoplasm of mammalian cells (Salerno et al., 2016). This novel technique has recently been used to show the intracellular delivery of several model cargos proteins (e.g. myoglobin, horseradish peroxidase, and ß-galactosidase) into a variety of cell lines (HEK and HT-3). We predict that the delivery of recombinant fluorescent KmUTAG will then allow us to visualize the distribution of SUMO and sumoylated proteins in living cells. The latter is of particular interest, because SUMO, proteins involved in SUMO dynamics, and certain SUMO-modified proteins are grossly increased or mislocalized in cell culture models of cancer [33,48], heart disease [49], viral infection [50], fertility-related problems [51], and neurodegenerative diseases [52].

## Materials and methods

### Strains and plasmids

The *K*. *marxianus* yeast strain BY28356 was purchased from the Yeast Genetic Resource Center Japan. Yeast strains for the two hybrid analysis as well as bacterial strains and plasmids for the production of MBP fusions proteins are described in Elmore et al., 2011. The SUMO-CAT expression plasmid was purchased as part of the Champion pET SUMO Expression system (Thermo Fisher, K30001). pEYFP-SUMO1 was purchased from addgene (#13380 [53]). Additional strains and plasmids are listed in the S2 Table.

### Web-based analyses and databases

The sequence alignment in Fig 1A was generated using T-Coffee and boxshade programs as described in https://labs.mcdb.ucsb.edu/weimbs/thomas/content/links. 3D structure of the ULP1 UD with Smt3 was derived using Cn3D software v4.3. Sequences analyzed in the S1 Table were compared using the *Kluyveromyces marxianus* DMKU3-1042 reference genome at www.ncbi.nlm.nih.gov/genome/10898, uniprot.org, and www.yeastgenome.org/. foldability indices were compared using http://bip.weizmann.ac.il/fldbin/findex.

### Cloning of KmUD and KmUTAG

Primers corresponding to the UD domain of kmULP1 (see Fig 1A) were used to amplify the KmUD coding sequence from genomic DNA of Km strain DMKU 3–1042. Mutagenic primers and the Q5 site-directed mutagenesis kit (NEB E0554S) were used to generate KmUTAG, KmUTAG^SIMΔ^, and ScUTAG^SIM^. To overexpress and purify Ulp1 truncations were cloned into pMALc-HT (a gift from Sean T Prigge, Department of Molecular Microbiology and Immunology, The Johns Hopkins University School of Public Health, Baltimore, MD, USA), thereby adding an in-frame MBP module followed by a TEV protease cleavage site and a His^6^ epitope tag (Elmore et al., 2011). AD-fusions of KmUD and kmUTAG were cloned into gapped pOAD2 (Stan Fields Lab, University of Washington, Seattle WA) and used for two-hybrid and complementation studies. All primer sequences are available upon request. Codon-optimized mCherry-tagged KmUTAG for expression in mammalian cells was cloned using a commercial service (Genewiz).

### Expression, purification, and *in vitro* assays

Frozen bacterial cell pellets from 100 ml of isopropyl β-D-1-thiogalactopyranoside-induced BL21 Star (DE3) cells were thawed on ice and resuspended in 2 ml of 1 × SUMO protease buffer (SPB: 50 mM Tris-HCl, pH 8.0, 0.2% NP-40, 150mM NaCl) containing 1 × TCEP (Tris(2-carboxyethyl)phosphine hydrochloride) (5mM) added just before use. Ice-cold cells were sonicated using a Branson Sonifier ultrasonic cell disruptor (Branson Ultrasonics, Danbury, CT, USA), and extracts were cleared by centrifugation at 15, 000 rpm for eight minutes at 4°C. Cleared bacterial extracts were added to 15-ml conical tubes and diluted using 4 ml of 1 × SPB containing 1 × TCEP. MBP/HIS6-tagged were bound to 5-ml columns containing 200 μl of amylose resin (New England Biolabs, Ipswich, MA, USA) or Talon Resin (Clontech #635502) and washed 3 times with 1 × cold SPB and then eluted with either 100mM maltose (amylose resin) or 20mM imidazole (Talon resin) in 1x SPB. SUMO-CAT was purified as per manufacture’s instructions (Thermo Fisher, K30001). Eluted proteins were supplemented with 10% Glycerol, supplemented with 5mM TCEP, and aliquoted before freezing in liquid nitrogen. For pulldown and binding reactions up to 6ug of each protein was added to dolphin-nose tubes containing 1ml of 1X SPB with or without TCEP as indicated and 20μl of SUMO beads were added. Reactions were nutated for 45 minutes or the indicated times, spun down, washed with 3 times with 1xSPB and then eluted with 40ul of SPB. To assess binding to soluble proteins such as SUMO1 and SUMO-CAT, 20ul of amylose resin was used to precipitate Talon-purified UD and UTAG fusions. 20μl of the reaction was run out on NUPAGE 4–12% Bis/Tris SDS PAGE gels. Gels were washed in water and then stained using Simply Blue G250 dye (Life technologies # LC6060) before scanning and quantitation using a BioRad imager and BioRad Image Lab software. SUMO protease digests were 20ul reactions containing 1xSPB with 5mM TCEP, serially diluted KmUD or KmUTAG (0.07μg, 0.007μg, 0.0007μg), and 6ug substrate. Reactions were incubated for 1 hour at 30°C and stopped with 20μl 2x SDS Page Loading buffer. Half of each reaction was run on NUPAGE 4–12% Bis/Tris before staining with Simply Blue dye.

### Biolayer interferometry

SUMO1 (Boston Biochem UL-712) was biotinylated using NHS-LC-LC-biotin (succini-midyl-6-[bio-tinamido]-6-hexanamidohexanoate) (Thermo Scientific) at a 5:1 molar ratio of biotin to protein for 30 min at 25°C followed by rapid exchange into HBS-T (10 mM HEPES, pH 7.4, 150 mM NaCl, 0.05% Tween 20) by passage over a rapid desalting column. Conditions were chosen according to the manufacturer so that each protein was likely randomly biotinylated at an average of 1–2 positions. All BLI measurements were made on a FortéBio (Menlo Park, CA) Octet QK biosensor using streptavidin sensors. Assays were performed in 96-well microplates at 25°C. All volumes were 200 μL. After loading biotinylated ligand onto SA sensors, a baseline was established in buffer alone prior to association at varying analyte concentrations for 300 s. After the association phase, sensors were moved to buffer only to monitor dissociation for another 300 s. Nonspecific binding to sensors without ligand was negligible. Reference subtracted raw data were fit with a single-state global association-then-dissociation model using GraphPad Prism 7.01.

### Antibodies, proteins, and other reagents

Bead conjugated or unconjugated pro-SUMO1, SUMO1, SUMO2 and SUMO3 were purchased from Boston Biochem. The Sumoylation kit for the productions and detection of sumoylated RG1 was purchased from Enzo Lifesciences (BML-UW8955-0001). Commercially available Ulp1 for in vitro desumoylation reactions was purchased from Thermo-Fisher (12588018). TCEP was purchased from Thermo Fisher (Pierce 120490). 10x Cell Lysis Buffer for cell extracts was purchased from Cell Signaling Technology (#9803) and cell extracts from mammalian cells were generated as recommended by the manufacturer. The anti GFP antibody used was JL-8 was from Takara (632381). The anti SUMO1 and SUMO2/3 antibodies were supplied with the Sumoylation kit.

## Supporting information

S1 Fig Analysis of SUMO1-binding when the putative core SIM in KmUTAG (VDIL) is replaced with TQID from ScUTAG. This mutant protein is labeled as UTAG*. The binding assay was performed as above and at room temperature.
(TIF)

S2 Fig Loading controls for Fig 3C. SUMO1 and proSUMO1 after a pulldown with kmUTAG (lane 1 and 2). SUMO1 and proSUMO1 input to the pulldown reaction (Lane 2 and 3). 20% of KmUTAG used as input to the pulldown reaction.
(TIF)

S1 Table Comparison of SUMO pathway components in *S*. *cerevisiae* (Sc) and *K*. *marxianus* (Km). Sequence comparisons were derived using: https://www.ncbi.nlm.nih.gov/genome/10898. FoldIndex comparisons (Δ) were calculating by substracting the reported Sc protein unfoldability score from the reported Km protein unfoldability score. (+) indicates that the Km protein is estimated to fold better (43).
(DOCX)

S2 Table Plasmids used in this study. (DOCX)

## References

[1] Kerscher O, Felberbaum R, Hochstrasser M. Modification of proteins by ubiquitin and ubiquitin-like proteins. Annu Rev Cell Dev Biol. 2006;22: 159–180. doi: 10.1146/annurev.cellbio.22.010605.093503 16753028 10.1146/annurev.cellbio.22.010605.093503

[2] Kerscher Oliver (2 2016) SUMOylation In: eLS. John Wiley & Sons, Ltd: Chichester doi: 10.1002/9780470015902.a0021849.pub2

[3] Da Silva-Ferrada E, Lopitz-Otsoa F, Lang V, Rodríguez MS, Matthiesen R. Strategies to Identify Recognition Signals and Targets of SUMOylation. Biochem Res Int. 2012;2012: 875148–875148. doi: 10.1155/2012/875148 22811915 10.1155/2012/875148 PMC3395311

[4] Xie Y, Kerscher O, Kroetz MB, McConchie HF, Sung P, Hochstrasser M. The yeast Hex3.Slx8 heterodimer is a ubiquitin ligase stimulated by substrate sumoylation. J Biol Chem. 2007;282: 34176–34184. doi: 10.1074/jbc.M706025200 17848550 10.1074/jbc.M706025200

[5] Uzunova K, Göttsche K, Miteva M, Weisshaar SR, Glanemann C, Schnellhardt M, et al Ubiquitin-dependent proteolytic control of SUMO conjugates. J Biol Chem. 2007;282: 34167–34175. doi: 10.1074/jbc.M706505200 17728242 10.1074/jbc.M706505200

[6] Prudden J, Pebernard S, Raffa G, Slavin DA, Perry JJP, Tainer JA, et al SUMO-targeted ubiquitin ligases in genome stability. EMBO J. 2007;26: 4089–4101. doi: 10.1038/sj.emboj.7601838 17762865 10.1038/sj.emboj.7601838 PMC2230673

[7] Perry JJP, Tainer JA, Boddy MN. A SIM-ultaneous role for SUMO and ubiquitin. Trends Biochem Sci. 2008;33: 201–208. doi: 10.1016/j.tibs.2008.02.001 18403209 10.1016/j.tibs.2008.02.001

[8] Sriramachandran AM, Dohmen RJ. SUMO-targeted ubiquitin ligases. Biochim Biophys Acta. 2014;1843: 75–85. doi: 10.1016/j.bbamcr.2013.08.022 24018209 10.1016/j.bbamcr.2013.08.022

[9] Hendriks IA, Vertegaal ACO. A comprehensive compilation of SUMO proteomics. Nat Rev Mol Cell Biol. 2016;17: 581–595. doi: 10.1038/nrm.2016.81 27435506 10.1038/nrm.2016.81

[10] Willson V. SUMO Regulation of Cellular Processes. Springer Science & Business Media; 2009.

[11] Li SJ, Hochstrasser M. A new protease required for cell-cycle progression in yeast. Nature. 1999;398: 246–251. doi: 10.1038/18457 10094048 10.1038/18457

[12] Li S-J, Hochstrasser M. The Ulp1 SUMO isopeptidase: distinct domains required for viability, nuclear envelope localization, and substrate specificity. J Cell Biol. 2003;160: 1069–1081. 12654900 10.1083/jcb.200212052 PMC2172760

[13] Li SJ, Hochstrasser M. The yeast ULP2 (SMT4) gene encodes a novel protease specific for the ubiquitin-like Smt3 protein. Mol Cell Biol. 2000;20: 2367–2377. 10713161 10.1128/mcb.20.7.2367-2377.2000 PMC85410

[14] Panse VG, Küster B, Gerstberger T, Hurt E. Unconventional tethering of Ulp1 to the transport channel of the nuclear pore complex by karyopherins. Nat Cell Biol. 2003;5: 21–27. doi: 10.1038/ncb893 12471376 10.1038/ncb893

[15] Kroetz MB, Su D, Hochstrasser M. Essential role of nuclear localization for yeast Ulp2 SUMO protease function. Mol Biol Cell. 2009;20: 2196–2206. doi: 10.1091/mbc.E08-10-1090 19225149 10.1091/mbc.E08-10-1090 PMC2669027

[16] Eckhoff J, Dohmen RJ. In Vitro Studies Reveal a Sequential Mode of Chain Processing by the Yeast SUMO (Small Ubiquitin-related Modifier)-specific Protease Ulp2. J Biol Chem. 2015;290: 12268–12281. doi: 10.1074/jbc.M114.622217 25833950 10.1074/jbc.M114.622217 PMC4424358

[17] Bylebyl GR, Belichenko I, Johnson ES. The SUMO isopeptidase Ulp2 prevents accumulation of SUMO chains in yeast. J Biol Chem. 2003;278: 44113–44120. doi: 10.1074/jbc.M308357200 12941945 10.1074/jbc.M308357200

[18] Makhnevych T, Ptak C, Lusk CP, Aitchison JD, Wozniak RW. The role of karyopherins in the regulated sumoylation of septins. J Cell Biol. 2007;177: 39–49. doi: 10.1083/jcb.200608066 17403926 10.1083/jcb.200608066 PMC2064105

[19] Elmore ZC, Donaher M, Matson BC, Murphy H, Westerbeck JW, Kerscher O. Sumo-dependent substrate targeting of the SUMO protease Ulp1. BMC Biol. 2011;9: 74–74. doi: 10.1186/1741-7007-9-74 22034919 10.1186/1741-7007-9-74 PMC3216068

[20] Kerscher O. SUMO junction-what’s your function? New insights through SUMO-interacting motifs. EMBO Rep. 2007;8: 550–555. doi: 10.1038/sj.embor.7400980 17545995 10.1038/sj.embor.7400980 PMC2002525

[21] de Albuquerque CP, Liang J, Gaut NJ, Zhou H. Molecular Circuitry of the SUMO (Small Ubiquitin-like Modifier) Pathway in Controlling Sumoylation Homeostasis and Suppressing Genome Rearrangements. J Biol Chem. 2016;291: 8825–8835. doi: 10.1074/jbc.M116.716399 26921322 10.1074/jbc.M116.716399 PMC4861450

[22] Hickey CM, Wilson NR, Hochstrasser M. Function and regulation of SUMO proteases. Nat Rev Mol Cell Biol. 2012;13: 755–766. doi: 10.1038/nrm3478 23175280 10.1038/nrm3478 PMC3668692

[23] Lewicki MC, Srikumar T, Johnson E, Raught B. The S. cerevisiae SUMO stress response is a conjugation-deconjugation cycle that targets the transcription machinery. J Proteomics. 2015;118: 39–48. doi: 10.1016/j.jprot.2014.11.012 25434491 10.1016/j.jprot.2014.11.012

[24] Srikumar T, Lewicki MC, Costanzo M, Tkach JM, van Bakel H, Tsui K, et al Global analysis of SUMO chain function reveals multiple roles in chromatin regulation. J Cell Biol. 2013;201: 145–163. doi: 10.1083/jcb.201210019 23547032 10.1083/jcb.201210019 PMC3613684

[25] Seifert A, Schofield P, Barton GJ, Hay RT. Proteotoxic stress reprograms the chromatin landscape of SUMO modification. Sci Signal. 2015;8: rs7–rs7. doi: 10.1126/scisignal.aaa2213 26152697 10.1126/scisignal.aaa2213 PMC6707813

[26] Schwartz DC, Felberbaum R, Hochstrasser M. The Ulp2 SUMO protease is required for cell division following termination of the DNA damage checkpoint. Mol Cell Biol. 2007;27: 6948–6961. doi: 10.1128/MCB.00774-07 17664284 10.1128/MCB.00774-07 PMC2099214

[27] Sydorskyy Y, Srikumar T, Jeram SM, Wheaton S, Vizeacoumar FJ, Makhnevych T, et al A novel mechanism for SUMO system control: regulated Ulp1 nucleolar sequestration. Mol Cell Biol. 2010;30: 4452–4462. doi: 10.1128/MCB.00335-10 20647537 10.1128/MCB.00335-10 PMC2937538

[28] Xu Z, Lam LSM, Lam LH, Chau SF, Ng TB, Au SWN. Molecular basis of the redox regulation of SUMO proteases: a protective mechanism of intermolecular disulfide linkage against irreversible sulfhydryl oxidation. FASEB J. 2008;22: 127–137. doi: 10.1096/fj.06-7871com 17704192 10.1096/fj.06-7871com

[29] Westerbeck JW, Pasupala N, Guillotte M, Szymanski E, Matson BC, Esteban C, et al A SUMO-targeted ubiquitin ligase is involved in the degradation of the nuclear pool of the SUMO E3 ligase Siz1. Mol Biol Cell. 2014;25: 1–16. doi: 10.1091/mbc.E13-05-0291 24196836 10.1091/mbc.E13-05-0291 PMC3873881

[30] Rojas-Fernandez A, Plechanovová A, Hattersley N, Jaffray E, Tatham MH, Hay RT. SUMO chain-induced dimerization activates RNF4. Mol Cell. 2014;53: 880–892. doi: 10.1016/j.molcel.2014.02.031 24656128 10.1016/j.molcel.2014.02.031 PMC3991395

[31] Mukhopadhyay D, Dasso M. Modification in reverse: the SUMO proteases. Trends Biochem Sci. 2007;32: 286–295. doi: 10.1016/j.tibs.2007.05.002 17499995 10.1016/j.tibs.2007.05.002

[32] Nayak A, Müller S. SUMO-specific proteases/isopeptidases: SENPs and beyond. Genome Biology. 2014;15: 422–422. doi: 10.1186/s13059-014-0422-2 25315341 10.1186/s13059-014-0422-2 PMC4281951

[33] Zhang H, Kuai X, Ji Z, Li Z, Shi R. Over-expression of small ubiquitin-related modifier-1 and sumoylated p53 in colon cancer. Cell Biochem Biophys. 2013;67: 1081–1087. doi: 10.1007/s12013-013-9612-x 23640307 10.1007/s12013-013-9612-x

[34] Wang Q, Xia N, Li T, Xu Y, Zou Y, Zuo Y, et al SUMO-specific protease 1 promotes prostate cancer progression and metastasis. Oncogene. 2013;32: 2493–2498. doi: 10.1038/onc.2012.250 22733136 10.1038/onc.2012.250

[35] Fu J, Yu H-MI, Chiu S-Y, Mirando AJ, Maruyama EO, Cheng J-G, et al Disruption of SUMO-specific protease 2 induces mitochondria mediated neurodegeneration. PLoS Genet. 2014;10: e1004579–e1004579. doi: 10.1371/journal.pgen.1004579 25299344 10.1371/journal.pgen.1004579 PMC4191884

[36] Zhang Q-S, Zhang M, Huang X-J, Liu X-J, Li W-P. Downregulation of SENP1 inhibits cell proliferation, migration and promotes apoptosis in human glioma cells. Oncol Lett. 2016;12: 217–221. doi: 10.3892/ol.2016.4558 27347128 10.3892/ol.2016.4558 PMC4907169

[37] Mossessova E, Lima CD. Ulp1-SUMO crystal structure and genetic analysis reveal conserved interactions and a regulatory element essential for cell growth in yeast. Mol Cell. 2000;5: 865–876. 10882122 10.1016/s1097-2765(00)80326-3

[38] Alegre KO, Reverter D. Swapping small ubiquitin-like modifier (SUMO) isoform specificity of SUMO proteases SENP6 and SENP7. J Biol Chem. 2011;286: 36142–36151. doi: 10.1074/jbc.M111.268847 21878624 10.1074/jbc.M111.268847 PMC3195590

[39] Yamamoto H, Shima T, Yamaguchi M, Mochizuki Y, Hoshida H, Kakuta S, et al The Thermotolerant Yeast Kluyveromyces marxianus Is a Useful Organism for Structural and Biochemical Studies of Autophagy. J Biol Chem. 2015;290: 29506–29518. doi: 10.1074/jbc.M115.684233 26442587 10.1074/jbc.M115.684233 PMC4705951

[40] Zhao Q, Xie Y, Zheng Y, Jiang S, Liu W, Mu W, et al GPS-SUMO: a tool for the prediction of sumoylation sites and SUMO-interaction motifs. Nucleic Acids Res. 2014;42: W325–30. doi: 10.1093/nar/gku383 24880689 10.1093/nar/gku383 PMC4086084

[41] Bailey D, O’Hare P. Characterization of the localization and proteolytic activity of the SUMO-specific protease, SENP1. J Biol Chem. 2004;279: 692–703. doi: 10.1074/jbc.M306195200 14563852 10.1074/jbc.M306195200

[42] Lertwattanasakul N, Kosaka T, Hosoyama A, Suzuki Y, Rodrussamee N, Matsutani M, et al Genetic basis of the highly efficient yeast Kluyveromyces marxianus: complete genome sequence and transcriptome analyses. Biotechnol Biofuels. 2015;8: 47 doi: 10.1186/s13068-015-0227-x 25834639 10.1186/s13068-015-0227-x PMC4381506

[43] Prilusky J, Felder CE, Zeev-Ben-Mordehai T, Rydberg EH, Man O, Beckmann JS, et al FoldIndex: a simple tool to predict whether a given protein sequence is intrinsically unfolded. Bioinformatics. 2005;21: 3435–3438. doi: 10.1093/bioinformatics/bti537 15955783 10.1093/bioinformatics/bti537

[44] Pinheiro R, Belo I, Mota M. Oxidative stress response of Kluyveromyces marxianus to hydrogen peroxide, paraquat and pressure. Appl Microbiol Biotechnol. 2002;58: 842–847. doi: 10.1007/s00253-001-0927-y 12021807 10.1007/s00253-001-0927-y

[45] Pinto MP, Carvalho AF, Grou CP, Rodríguez-Borges JE, Sá-Miranda C, Azevedo JE. Heat shock induces a massive but differential inactivation of SUMO-specific proteases. Biochim Biophys Acta. 2012;1823: 1958–1966. doi: 10.1016/j.bbamcr.2012.07.010 22867988 10.1016/j.bbamcr.2012.07.010

[46] Psakhye I, Jentsch S. Protein group modification and synergy in the SUMO pathway as exemplified in DNA repair. Cell. 2012;151: 807–820. doi: 10.1016/j.cell.2012.10.021 23122649 10.1016/j.cell.2012.10.021

[47] Nie M, Boddy MN. Pli1PIAS1 SUMO Ligase Protected by the Nuclear Pore-associated SUMO Protease Ulp1SENP1/2. Journal of Biological Chemistry. 2015;290: 22678–22685. doi: 10.1074/jbc.M115.673038 26221037 10.1074/jbc.M115.673038 PMC4566240

[48] Guo W-H, Yuan L-H, Xiao Z-H, Liu D, Zhang J-X. Overexpression of SUMO-1 in hepatocellular carcinoma: a latent target for diagnosis and therapy of hepatoma. J Cancer Res Clin Oncol. 2011;137: 533–541. doi: 10.1007/s00432-010-0920-x 20502916 10.1007/s00432-010-0920-x PMC11957374

[49] Boudreau É, Labib S, Bertrand AT, Decostre V, Bolongo PM, Sylvius N, et al Lamin A/C mutants disturb sumo1 localization and sumoylation in vitro and in vivo. PLoS One. 2012;7: e45918–e45918. doi: 10.1371/journal.pone.0045918 23029315 10.1371/journal.pone.0045918 PMC3448699

[50] McNally BA, Trgovcich J, Maul GG, Liu Y, Zheng P. A role for cytoplasmic PML in cellular resistance to viral infection. PLoS One. 2008;3: e2277 doi: 10.1371/journal.pone.0002277 18509536 10.1371/journal.pone.0002277 PMC2386554

[51] Vigodner M, Shrivastava V, Gutstein LE, Schneider J, Nieves E, Goldstein M, et al Localization and identification of sumoylated proteins in human sperm: excessive sumoylation is a marker of defective spermatozoa. Hum Reprod. 2013;28: 210–223. doi: 10.1093/humrep/des317 23077236 10.1093/humrep/des317 PMC3522414

[52] Kim YM, Jang WH, Quezado MM, Oh Y, Chung KC, Junn E, et al Proteasome inhibition induces α-synuclein SUMOylation and aggregate formation. J Neurol Sci. 2011;307: 157–161. doi: 10.1016/j.jns.2011.04.015 21641618 10.1016/j.jns.2011.04.015 PMC3129438

[53] Ayaydin F, Dasso M. Distinct in vivo dynamics of vertebrate SUMO paralogues. Mol Biol Cell. 2004;15: 5208–5218. doi: 10.1091/mbc.E04-07-0589 15456902 10.1091/mbc.E04-07-0589 PMC532004

