# Supplementary figures and images for: SUMO targeting of a stress-tolerant Ulp1 SUMO protease
Source: PLoS One. 2018 Jan 19;13(1):e0191391. doi: 10.1371/journal.pone.0191391 (PMC5774762; doi:10.1371/journal.pone.0191391)

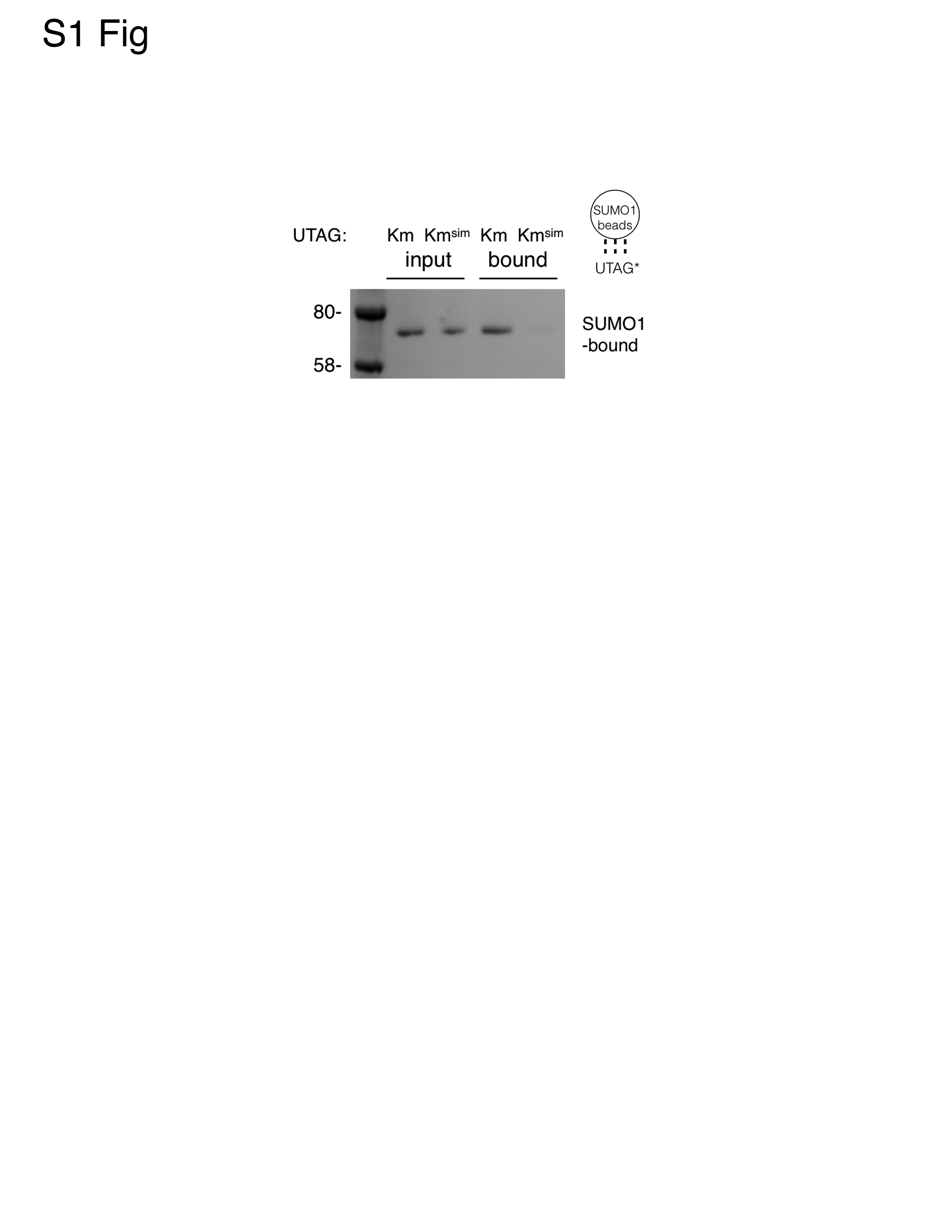

Supplement: S1 Fig — This mutant protein is labeled as UTAG*. The binding assay was performed as above and at room temperature. (TIF) [file pone.0191391.s001.tif]

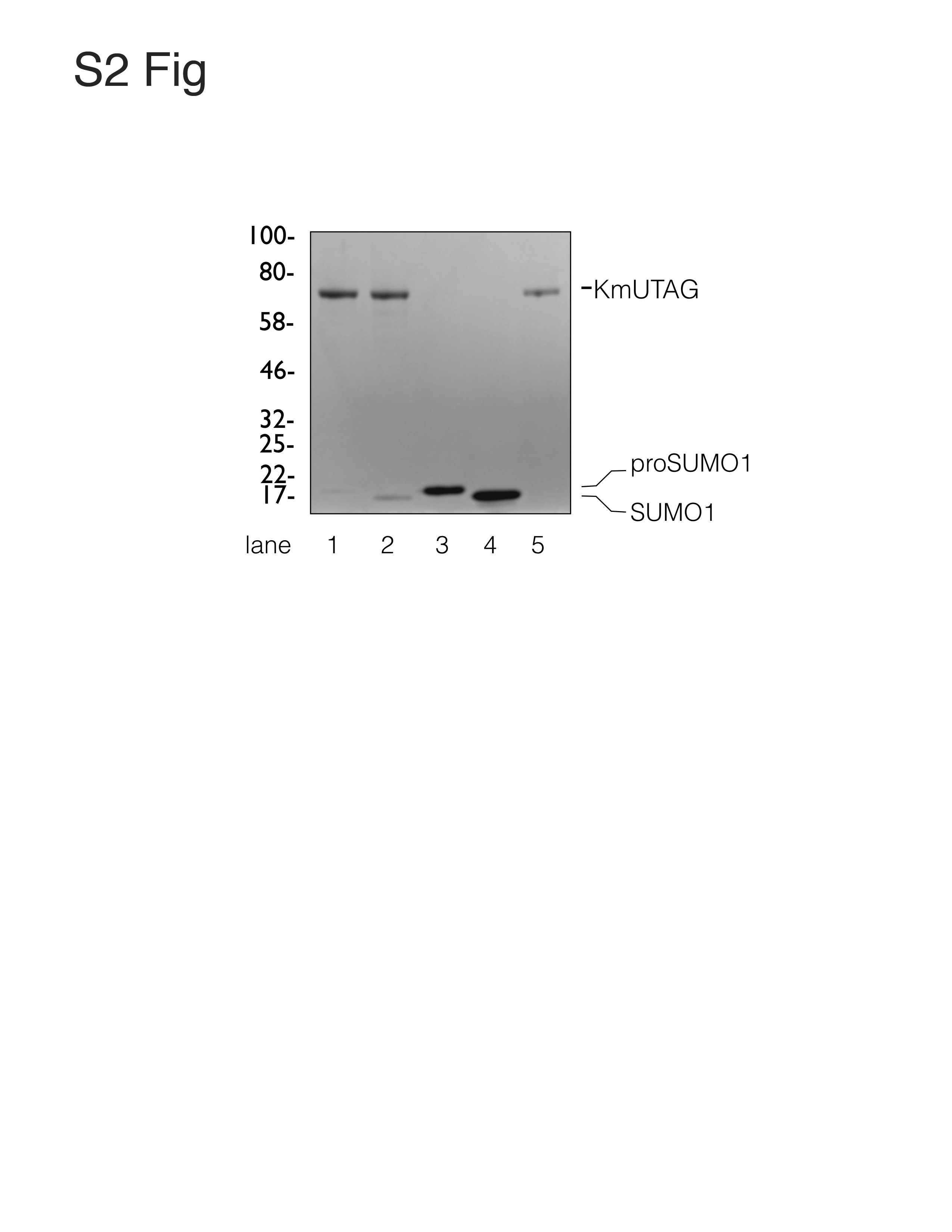

Supplement: S2 Fig — SUMO1 and proSUMO1 after a pulldown with kmUTAG (lane 1 and 2). SUMO1 and proSUMO1 input to the pulldown reaction (Lane 2 and 3). 20% of KmUTAG used as input to the pulldown reaction. (TIF) [file pone.0191391.s002.tif]
