# Supplementary material for: SUMO targeting of a stress-tolerant Ulp1 SUMO protease
Source: PLoS One. 2018 Jan 19;13(1):e0191391. doi: 10.1371/journal.pone.0191391 (PMC5774762; doi:10.1371/journal.pone.0191391)
Supplement: S1 Table — Sequence comparisons were derived using: https://www.ncbi.nlm.nih.gov/genome/10898. FoldIndex comparisons (Δ) were calculating by substracting the reported Sc protein unfoldability score from the reported Km protein unfoldability score. (+) indicates that the Km protein is estimated to fold better (43). (DOCX) [file pone.0191391.s003.docx]

S1 table:

Comparison of SUMO pathway components in *S. cerevisiae* (Sc) and *K. marxianus* (Km)

| Sc protein | Km locus tag/protein name; protein product | Km vs. Sc (aa) length & (difference) | Identity  (aa) | Query coverage | E-value | ∆ in FoldIndex ** |
| --- | --- | --- | --- | --- | --- | --- |
| Smt3 | KLMA_10072/Smt3;  [BAO37694.1](https://www.ncbi.nlm.nih.gov/protein/BAO37694.1) | 93:101  (+8) | 85% | 93% | 2.1e-46 | +0.008 |
| Ulp1 | KLMA_20214/Ulp1;  [BAO38672.1](https://www.ncbi.nlm.nih.gov/protein/BAO38672.1) | 555:621  (+66) | 51% | 74% | 5e-145 | + 0.085 |
| Ulp2 | KLMA_40345/Ubiquitin-like-specific protease 2; [BAO40369.1](https://www.ncbi.nlm.nih.gov/protein/BAO40369.1) | 1048:1034  (+14) | 44% | 34% | 4e-87 | - 0.068 |
| Ubc9 | KLMA_70064, SUMO-conjugating enzyme UBC9  ; [BAO41912.1](https://www.ncbi.nlm.nih.gov/protein/BAO41912.1) | 157:157  (0) | 83% | 100% | 3e-99 | - 0.009 |
| Aos1 | KLMA_20499/DNA damage tolerance protein RHC31;  [BAO38957.1](https://www.ncbi.nlm.nih.gov/protein/BAO38957.1) | 337:347  (10) | 55% | 100% | 3e-131 | + 0.14 |
| Uba2 | KLMA_20577/ubiquitin-activating enzyme E1-like; [BAO39035.1](https://www.ncbi.nlm.nih.gov/protein/BAO39035.1) | 631:636  (5) | 62% | 85% | 0.0 | + 0.04 |
| Siz1 | KLMA_30651/e3 SUMO-protein ligase SIZ1; [BAO39946.1](https://www.ncbi.nlm.nih.gov/protein/BAO39946.1) | 805:904  (99) | 43% | 65% | 2e-115 | + 0.108 |
| Siz2 | No Km ortholog found | xxx:726 |  |  |  |  |
| Mms21 | KLMA_10563/e3 SUMO-protein ligase MMS21  Mms21; [BAO38185.1](https://www.ncbi.nlm.nih.gov/protein/BAO38185.1) | 254:267  (13) | 29% | 97% | 1e-36 | + 0.019 |
| Slx5 | KLMA_10201/ e3 ubiquitin-protein ligase complex SLX5-SLX8 subunit SLX5  ; [BAO37823.1](https://www.ncbi.nlm.nih.gov/protein/BAO37823.1) | 612:619  (7) | 39% | 99% | 1e-120 | - 0.016 |
| Slx8 | KLMA_70156/ conserved hypothetical protein; [BAO42004.1](https://www.ncbi.nlm.nih.gov/protein/BAO42004.1) | 271:274  (3) | 48% | 35% | 9e-25 | -0.055 |

Km strain: DMKU3-1042

• Km sequences from: <https://www.ncbi.nlm.nih.gov/genome/10898>

• aa = amino acids

** <http://bip.weizmann.ac.il/fldbin/findex> -- (+) indicates that the Km protein is estimated to fold better.

FoldIndex: a simple tool to predict whether a given protein sequence is intrinsically unfolded. Prilusky J, Felder CE, Zeev-Ben-Mordehai T, Rydberg EH, Man O, Beckmann JS, Silman I, Sussman JL.

Bioinformatics. 2005 Aug 15;21(16):3435-8. PMID:15955783
