# Supplementary material for: SUMO targeting of a stress-tolerant Ulp1 SUMO protease
Source: PLoS One. 2018 Jan 19;13(1):e0191391. doi: 10.1371/journal.pone.0191391 (PMC5774762; doi:10.1371/journal.pone.0191391)
Supplement: S2 Table — (DOCX) [file pone.0191391.s004.docx]

S2 table

Plasmids used in this study

| ID# | Plasmid name | Plasmid backbone | marker | Reference / vendor |
| --- | --- | --- | --- | --- |
| BOK1424 | pET SUMO/CAT | pET SUMO | Kan | Thermo Fisher K30001 |
| BOK1418 | KmUTAG^SIM^ putative SIM replaced with corresponding sequence in ScUlp1 in pMALc-HT | pMALc-HT | amp | This work |
| BOK1382 | KmUD | pMALc-HT | amp | This work |
| BOK1381 | KmUTAG | pMALc-HT | amp | This work |
| BOK752 | ScUTAG | pMALc-HT | amp | Elmore et al., 2011 |
| BOK651 | ScUD | pMALc-HT | amp | This work |
| BOK1357 | AD-KmUD | pOAD | LEU2/CEN amp | This work |
| BOK1358 | AD-KmUTAG | pOAD | LEU2/CEN amp | This work |
| BOK1354 | AD-ScUD | pOAD | LEU2/CEN amp | This work |
| BOK1355 | AD-ScUTAG | pOAD | LEU2/CEN amp | This work |
